# Cognitive Deficit in Schizophrenia: From Etiology to Novel Treatments

**DOI:** 10.3390/ijms22189905

**Published:** 2021-09-14

**Authors:** Antón L. Martínez, José Brea, Sara Rico, María Teresa de los Frailes, María Isabel Loza

**Affiliations:** 1 BioFarma Research Group, Centro Singular de Investigación en Medicina Molecular y Enfermedades Crónicas (CIMUS), Universidade de Santiago de Compostela, 15782 Santiago de Compostela, Spain; antonleandro.martinez@usc.es (A.L.M.); pepo.brea@usc.es (J.B.); sararicolopez19@gmail.com (S.R.); 2 Experimental Sciences Faculty, Universidad Francisco de Vitoria, 28223 Pozuelo de Alarcón, Spain; maite.delosfrailes@ufv.es

**Keywords:** schizophrenia, cognitive deficit, antipsychotics, pharmacology, mechanisms of action

## Abstract

Schizophrenia is a major mental illness characterized by positive and negative symptoms, and by cognitive deficit. Although cognitive impairment is disabling for patients, it has been largely neglected in the treatment of schizophrenia. There are several reasons for this lack of treatments for cognitive deficit, but the complexity of its etiology—in which neuroanatomic, biochemical and genetic factors concur—has contributed to the lack of effective treatments. In the last few years, there have been several attempts to develop novel drugs for the treatment of cognitive impairment in schizophrenia. Despite these efforts, little progress has been made. The latest findings point to the importance of developing personalized treatments for schizophrenia which enhance neuroplasticity, and of combining pharmacological treatments with non-pharmacological measures.

## 1. Introduction

Schizophrenia is a major mental illness characterized by psychosis, apathy, social withdrawal and cognitive impairment [1]. The symptoms of schizophrenia have been placed into three categories: positive symptoms such as delusions and hallucinations [2]; negative symptoms such as affective flattening, alogia and diminished emotional expression; and cognitive deficit [3]. Although schizophrenia was considered an early dementia in the 19th century, the cognitive symptoms of schizophrenia have been largely neglected in the treatment of the disease [4]. However, in the last few years, there has been a growing interest in the study of cognitive impairment [5]. The reasons for this are that cognitive impairment is one of the first symptoms to manifest in schizophrenia, it is disabling for schizophrenic patients, and it contributes to their functional impairment [6]. Cognitive deficit cannot be treated with current antipsychotic drugs, which can only effectively treat positive symptoms of the disease [7]. Therefore, cognitive impairment in schizophrenia is still a clear unmet clinical need.

Cognitive deficit comprises an impairment in several cognitive domains, such as processing speed, attention, working memory, verbal and visual learning, problem solving and social cognition [8]. This impairment is found even in the first episode of schizophrenia [9].

Cognitive deficit is a process involving genomic, neurobiological and neuroanatomic factors that interact with each other in a complex way. The aim of this review is to go over the etiology of neurocognitive impairment in schizophrenia and the status of current research on the treatments for this symptom of schizophrenia.

## 2. Etiology of Neurocognitive Impairment

### 2.1. Neuroanatomic Findings

Several image studies have described that cognitive deficit in schizophrenia is associated with cortical thickness [10,11,12]. Interestingly, this effect is more pronounced in women than in men, where there may not even be a significant decrease in cortical thickness [13]. Indeed, cognitive deficit is related to other changes in brain structure and function, such as a greater ventricular volume [14], a reduction in cerebellar volume [13], a decrease in the function of basal ganglia [15] and a loss of dendritic spines in pyramidal neurons of the dorsolateral prefrontal cortex (DLPFC) [16]. These changes could be related to the observed disruption in cortico-cerebellar-thalamic-cortical circuits in patients suffering from schizophrenia [17], and to the reduction in the metabolic rate of the prefrontal cortex [18].

Similar neuroanatomic changes have also been observed in animal models of schizophrenia [19] and, interestingly, in patients suffering from encephalitis caused by herpes virus [20].

Changes in brain neuroanatomy used to be attributed to disorders in neurodevelopment [21,22], but it is now assumed that these disorders do not explain the whole process and it is believed that cognitive impairment might be due to the cumulative effect of neurodevelopmental abnormalities, changes in neuronal maturation and alterations in neuroplasticity [23].

### 2.2. Biochemical Findings

Cognitive deficit in schizophrenia has been related to an increase in inflammatory cytokines, to an imbalance in hormones such as cortisol and prolactin, in neurotrophic factors such as BDNF and in neurotransmitters such as GABA and glutamate [24].

#### 2.2.1. Inflammatory Cytokines

It has been observed that an increase in blood levels of C-reactive protein (CRP) is related to a lower cognitive performance, especially in verbal management, visual and working memory, processing speed, problem solving, executive function speed and in attention according to the meta-analysis by Bora et al. [5]. Interestingly, reductions in CRP levels are associated with an improvement in cognitive function [25].

Although the evidence is weaker than for CRP, an increase in other inflammatory cytokines such as IL-1ß and IL-6 has been also observed in schizophrenic patients with cognitive deficit [26,27]. These findings are in accordance with several studies that relate inflammation and neurodegeneration [28].

#### 2.2.2. Hormones

Cognitive deficit in schizophrenic patients has been linked to imbalances in serum levels of cortisol and prolactin.

Cortisol is a hormone that participates in the response to stress and inflammation. Moreover, cortisol easily crosses the blood–brain barrier, binding glucocorticoid receptors in prefrontal cortex, hippocampus and amygdala [29]. Higher cortisol levels are associated with lower hippocampal volume and lower BDNF expression [30,31]. Additionally, higher levels of cortisol and greater blunting of the cortisol awakening response (CAR) are related to poorer performance in cognitive tasks [32,33]. However, the influence of cortisol levels on neuroanatomic findings and in cognitive performance may depend on the sex of the patients and the clinical diagnosis of the patient [34].

Prolactin levels have also been linked to cognitive impairment. However, the study of prolactin levels in schizophrenic patients is difficult because typical antipsychotics, as D_2_ receptor antagonists, increase prolactin concentrations in blood because they block D_2_ receptors in the tuberoinfundibular pathway [35]. It has been observed that in antipsychotic-naive schizophrenia patients, higher levels of prolactin are related to poorer cognitive performance [36]. The reasons for this poorer performance are not clear, but it was observed that there is a decrease in grey matter volume in patients with hyperprolactinemia [37], and that prolactin levels are related to higher inflammatory markers [38].

#### 2.2.3. Neurotrophic Factors

BDNF is a neuropeptide that enhances brain remodeling and synaptic plasticity. It was observed that reduced levels of BDNF are related to lower cognitive capacities in patients with schizophrenia [39,40]. This reduction in BDNF levels has also been observed in other pathologies related to cognitive deficit. Vasconcelos et al. observed that this reduction in BDNF is related to an increase in oxidative stress in schizophrenic patients, as the administration of alpha-lipoic acid, a drug that counteracts free radicals, increases BDNF and improves cognitive capacities in animal models of schizophrenia [41]. Indeed, clozapine, an antipsychotic drug that improves negative symptoms and cognitive capacities in schizophrenic patients, increases brain BDNF levels [42]. The influence of variants of BDNF in cognitive deficit has also been studied; one example is BDNF Val66Met, which is related to a worse cognitive function [43,44]. However, there is a great deal of variability between different studies because of the heterogeneity among ethnic groups.

#### 2.2.4. Neurotransmitters

Cognitive deficit in schizophrenia has been linked to imbalances in neurotransmitters such as glutamate, GABA, dopamine, acetylcholine and histamine [45]. Glutamate and GABA play a major and well-known role in cognitive deficit in schizophrenia. An imbalance in both neurotransmitters was observed in DLPFC [46]. This area is associated with working memory—that is, the ability to manipulate information to guide behavior or thought [47]. The performance of working memory tasks implies the activation of the area showing an increase in gamma oscillatory activity (30–80 Hz) of glutamatergic pyramidal neurons, whose synchrony is regulated by GABA and glutamate [48]. In patients with schizophrenia-related cognitive deficit, there is no increase in gamma oscillatory activity in response to working memory demands [49].

It has been described that in patients suffering from schizophrenia-induced cognitive deficit there is a reduction in the glutamatergic stimulation of NMDA receptors in pyramidal neurons [50]. The activation of NMDA receptors depends upon the binding of co-agonists such as D-serine and glycine [51]. In schizophrenia-induced cognitive deficit there is a higher blockage of glycine sites in NMDA receptors due to higher levels of kynurenic acid in dorsal prefrontal cortex [52]. Inflammatory cytokines have also been reported to increase kynurenic acid levels, linking the observed increase in inflammatory cytokines with imbalances in glutamate signaling in schizophrenia subjects [53]. This is in agreement with the fact that most animal models of schizophrenia implicate the administration of drugs that block NMDA receptors to the animals, such as MK-801 (dizocilpine), ketamine or PCP (phencyclidine) [54].

However, the reduced sensitivity of NMDA receptors in pyramidal neurons in schizophrenia patients does not implicate a reduction in glutamate in DLPFC [55]. In fact, it has been described that glutaminase, the enzyme that participates in glutamate synthesis, is highly overexpressed in schizophrenia subjects [56]. Furthermore, there is an increase in the expression of metabotropic glutamate receptors in DLPFC of schizophrenia patients [57]. Those receptors exert a presynaptic regulation of glutamatergic signaling [58].

These imbalances in glutamatergic signaling are accompanied by changes in GABAergic inputs to pyramidal neurons, which are also crucial for pyramidal neurons’ synchronicity [59]. It was observed that in schizophrenia subjects there is a reduction in the expression of the 67 kDa isoform of glutamate decarboxylase (GAD67) [60]. This enzyme synthetizes GABA, and its deficit suggests a reduction in GABAergic signaling in DLPFC. This is consistent with the observed reduction in parvalbumin (PV) in DLPFC [61]. PV is a marker of GABAergic interneurons on DLPFC that buffers calcium facilitating GABA release [62], which modulates the activity of pyramidal neurons [63]. The reduction in PV suggests that there is a reduction in GABA-mediated synapsis.

There is also a reduction in other subpopulations of GABAergic neurons, such as somatostatin (SST)-expressing GABAergic neurons. A global reduction in SST expression in DLPFC in schizophrenia patients has been reported [64].

Although not fully described, changes in other neurotransmitters such as acetylcholine, histamine or dopamine have been reported in schizophrenia-induced cognitive deficit patients [46]. It has been suggested that there is a relationship between a decrease in striatal dopamine and the observed disruption in cortico-cerebellar-thalamic-cortical circuits in cognitive deficits in schizophrenia [65,66].

Recently, it has been demonstrated that the acetylcholinesterase blocker galantamine improves cognitive deficit in schizophrenia, suggesting that a reduction in acetylcholine signaling may play a role in cognitive deficit [67]. It has also been observed that, in schizophrenic patients, there is lower M_1_ receptor binding in the hippocampus, which results in impaired learning [68].

The role of histamine in cognitive deficit in schizophrenic patients is unclear, but Jin et al. described an increase in the expression of histamine H_3_ receptors in prefrontal cortex neurons of schizophrenic patients. These data suggest that the H_3_ receptor may play a role in cognitive decline in those patients [69].

### 2.3. Genetic Findings

Schizophrenia is a multifactorial disease whose etiology involves the interaction between environmental and genetic factors (see [70] for a review). Several genes related to cognitive deficit in schizophrenia have been identified.

Most of them encode proteins that participate in neurotransmission, such as the glutamate receptor-encoding *GRIN2B* and *GRIN2A* genes, the serotonin receptor-encoding *HTR2A* gene or the *COMT* gene [70]. *COMT* encodes catechol-O-methyltransferase, the enzyme that degrades catecholamines such as noradrenaline and dopamine [71]. Val158Met *COMT* polymorphism has been related to social cognitive deficits [72], resulting from a reduction in dopaminergic neurotransmission [73]. Nevertheless, more research is needed to identify the exact role of *COMT* polymorphisms in schizophrenia. Another gene involved in cognitive deficit in schizophrenia is *AKT1*, which encodes a serine threonine kinase activated by dopamine type 2 receptor agonists [74]. It also plays a crucial role in the neuregulin signaling pathway, and is related with social recognition [75].

Another subset of genes involved in schizophrenia are those related to brain development, for example *DTNBP1*, encoding dysbindin. This protein is involved in hippocampal formation [76], highlighting its role in cognitive processes critical to schizophrenia [77]. In recent years, several studies have associated the presence of single-nucleotide polymorphisms (SNPs) with cognitive deficit in schizophrenia [78,79]. Another gene related to neurodevelopment is *DISC1*, which participates in the acquisition of neuronal phenotypic features including axonal growth and dendritic spine formation, and in neuronal intracellular transport [80]. It was observed that some variants of the *DISC1* gene are associated with alterations in the expression of genes that participate in brain development, leading to intellectual disabilities [81].

## 3. Treatment of Cognitive Deficit in Schizophrenia

The treatment of schizophrenia currently relies on antipsychotics. There are two different subgroups of antipsychotics: typical and atypical [82]. Typical antipsychotics such as haloperidol or chlorpromazine are D_2_ receptor antagonists [83]. They are effective against positive symptoms, but they are less effective and even ineffective against negative symptoms and cognitive deficit [83]. Atypical antipsychotics like clozapine, olanzapine and risperidone block both D_2_ and 5-HT_2A_ receptors, modulating both glutamatergic and dopaminergic neurotransmission in prefrontal cortex [84].

Several meta-analyses have reflected that atypical antipsychotics elicited a slight improvement in some cognitive functions, while none of them were found to have a positive profile in all cognitive functions [85,86]. In the most recent study, Baldez et al. analyzed 54 randomized double-blinded studies to create a ranking of the effect of antipsychotics on neuropsychological tests [87]. They found that olanzapine was ranked first in motor performance and visuoconstruction, amisulpride came first in attention and verbal memory, ziprasidone in working memory, sertindole in processing speed and perphenazine in executive functions, while lurasidone occupied the first position in a composite score. They also observed that clozapine and typical antipsychotics occupied the last positions [87].

De la Fuente-Revenga et al. reported that chronic treatments with atypical antipsychotics such as clozapine could induce the transcription of the HDAC2 gene, with a detrimental effect on cognitive functions in cortical neurons [88]. This effect seems to be mediated by 5-HT_2A_ receptors [89], suggesting that compensatory mechanisms may confer atypical antipsychotics both positive and negative effects on cognitive function, with a high variability between individuals. Therefore, because the efficacy of antipsychotics in treating cognitive deficit is very poor, several strategies have been developed to address the cognitive symptoms of schizophrenia (Table 1).

### 3.1. Antioxidant Compounds

A multitude of drugs with antioxidant effects have been tested. In clinical trials, they appeared to be effective in alleviating cognitive deficit in schizophrenia, although most trials included a low number of patients [127].

Some antioxidant compounds have been reported to induce the transcription of genes encoding neurogenesis-related proteins [128]. Thus, alpha-lipoic acid increases BDNF expression in animal models of schizophrenia, with an improvement in memory impairment [41]. In a small clinical trial, it was observed that dietary supplementation with n-3 polyunsaturated fatty acids (PUFAs) counteracted cortical thickness observed in schizophrenic patients [90]. Another effect of antioxidant compounds is the enhancement of the nitrergic activity in the central nervous system [41]. Nitric oxide has been demonstrated to be effective in activating NMDA receptors in animal models of schizophrenia [129], but its efficacy has not yet been confirmed in clinical trials [130].

Another antioxidant compound, N-acetylcysteine, has been described as slightly beneficial for cognitive deficit in schizophrenic patients, improving cognitive speed [91]. This may be due to its role as precursor of glutamate, enhancing glutamatergic transmission in DLPFC and complementing its antioxidant effect [131,132].

Tetracyclines are a group of antibiotic drugs with an antioxidant effect that have been suggested to be beneficial for cognitive deficit in schizophrenia [133]. In clinical trials, minocycline has demonstrated a beneficial effect in alleviating cognitive deficit in schizophrenia, improving information processing speed [92,93].

In summary, it has been observed that drugs with an antioxidant effect may help to relieve cognitive deficit in schizophrenia by a combination of neurogenic, neuroprotective and nitrergic mechanisms, but their role in the pharmacotherapy of schizophrenic patients has not yet been properly established.

### 3.2. Modulation of Serotonergic Neurotransmission

As described above, atypical antipsychotic drugs interact with serotonin 5-HT_2A_ receptors, with both positive and negative effects on cognitive function. The negative effects of chronic antipsychotic treatments on cognitive function mediated by 5-HT_2A_ receptors could be avoided employing HDAC2 inhibitors [134].

In addition to the role of 5-HT_2A_ receptors in the treatment of cognitive deficit in schizophrenic patients, the role of other serotonin receptors has also been studied. Some atypical antipsychotic drugs, such as quetiapine or aripiprazole, stimulate 5-HT_1A_ receptors [135,136], providing an inhibitory feedback control of serotonin release in several areas of the brain [137]. This effect is related with an improvement in some cognitive functions such as verbal memory in schizophrenic patients, as seen with the 5-HT_1A_ agonist tandospirone [138]. Nevertheless, activation of 5-HT_1A_ receptors is also associated with hallucinations and nightmares, suggesting that 5-HT_1A_ receptor agonists may exacerbate positive symptoms of schizophrenia [139].

Antagonists of 5-HT_3_ receptors, such as the anti-emetic drug ondansetron, were shown to be mildly effective against cognitive deficits in schizophrenia in clinical trials, although these trials were conducted with very few patients [95]. Tropisetron, another 5-HT_3_ receptor antagonist, improved cognition in schizophrenic patients [96]. Tropisetron is also an agonist of α_7_ nicotinic acetylcholine receptors, demonstrating that the synergy between acetylcholine receptor agonism and 5-HT_3_ receptor antagonism is beneficial for the treatment of cognitive deficit in schizophrenia [140].

The 5-HT_6_ receptor is another serotonin receptor involved in cognitive function (see [141] for a review). It was demonstrated that AVN-492, as an antagonist of 5-HT_6_ receptors, counteracted cognitive impairment in animal models of schizophrenia [142]. Nevertheless, findings in clinical trials with schizophrenic patients were contradictory. Only some of them showed a scarce beneficial effect of the 5-HT_6_ antagonist AVN-211 in cognitive function [97,98]. This may be due to the fact that most of the compounds assayed as cognitive enhancers were not specific 5-HT_6_ receptor antagonists, suggesting the existence of synergistic interactions with other receptors.

The 5-HT_7_ receptor has also been proposed as a target for novel drugs improving cognitive enhancement (see [143] for a review). It has been suggested that the activation of the 5-HT_7_ receptor reduces neuronal excitability, and it has been proposed that 5-HT_7_ antagonists could exert a beneficial effect in cognition and memory [144]. In animal models of schizophrenia, it has been demonstrated that the inhibition of 5-HT_7_ receptors exerts a synergistic effect with the inhibition of other receptors such as sigma receptors or 5-HT_1_ receptors, both by combining them with an atypical antipsychotic or by using a multitarget strategy [145,146,147].

Although there is not much information about 5-HT_5A_ receptor function, it has been demonstrated that its inhibition exerts a procognitive effect in animal models of schizophrenia [148,149]. A recent work by Yamazaki et al. demonstrated that this effect is due to the activation of dopaminergic and GABAergic neurons in the prefrontal cortex as a result of the inhibition of the serotonin receptor [150].

Some antidepressants are also modulators of serotonergic neurotransmission, as serotonin reuptake inhibitors. However, recent evidence indicates that their effect may be related to an enhancement of neuroplasticity [151].

### 3.3. Regulation of GABAergic Neurotransmission

As described above, there is a reduction in the GABAergic transmission in DLPFC in schizophrenic patients, leading to a desynchronization of the depolarization of pyramidal neurons. Nevertheless, benzodiazepines, as non-selective agonists of GABA receptors, exert a deleterious effect in cognitive function in schizophrenic patients—specifically in attention and working memory [99]. This may be due to the fact that stimulation of GABA receptors exerts contradictory actions in neurons. The stimulation of synaptic GABA_A_ receptors induces a phasic inhibition of the neuron, and the stimulation of extrasynaptic GABA_A_ receptors induces a tonic inhibition of the neuron [7]. Attempts to develop selective modulators of GABA_A_ receptors did not yield compounds with a procognitive effect [152].

On the other hand, one GABA_B_ receptor agonist, baclofen, showed an improvement of cognitive function in an animal model of schizophrenia [153]. However, this result contradicts those published in other studies [7].

Another strategy to regulate GABAergic neurotransmission is the employment of GABA prodrugs such as BL-1020, an ester between the atypical antipsychotic perphenazine and GABA [154]. The use of this prodrug demonstrated a beneficial effect on cognitive function in animal models of schizophrenia and in phase 2 clinical trials [100,155]. Unfortunately, the results of phase 3 assays were negative [101].

### 3.4. Potentiation of Histaminergic Neurotransmission

An increase in the expression of histamine H_3_ receptors in the prefrontal cortex has been observed in schizophrenic patients. In fact, it has been observed that H_3_ receptor antagonists exerted a procognitive role in preclinical cognitive models [156]. However, in a phase 2 clinical trial, the H_3_ receptor antagonist ABT-288 failed to demonstrate any benefit in comparison to placebo in schizophrenic patients [102]. Recent research has described a procognitive effect of samelisant, an inverse agonist of H_3_ receptors, in animal models of schizophrenia [157]. Nevertheless, more studies are required.

### 3.5. Potentiation of Cholinergic Neurotransmission

Smoking has long been observed to be more common in schizophrenic patients than in the general population [158]. It has been proposed that this could be due to the fact that nicotine improves cognition in schizophrenic patients [159,160]. Indeed, it has been widely described that α_7_ nicotinic acetylcholine receptor activators have a beneficial procognitive effect both in animal models of schizophrenia [161,162] and in patients, although modulation of nicotinic receptors in humans entails a high risk of adverse events [163]. The reason for the beneficial effect of α_7_ nicotinic acetylcholine receptor activators on cognitive deficit is complex. On the one hand, they enhance theta activity and synaptic plasticity in hippocampal neurons, potentiating memory [164]. On the other hand, a nicotinic acetylcholine receptor agonist was shown to enhance the release of dopamine, glutamate and acetylcholine in the cerebral cortex and in the nucleus accumbens, which are reduced in schizophrenic patients with cognitive deficit [165].

One of the assayed α_7_ nicotinic acetylcholine receptor activators is varenicline, which is widely used for smoking cessation [166]. Although a beneficial effect was observed in animal models [167,168], there is no evidence from clinical trials of its efficacy in alleviating cognitive deficit in schizophrenic patients [103]. Other compounds activating α_7_ nicotinic acetylcholine receptors such as bradanicline, nelonicline and encenicline did not show a beneficial effect in clinical trials [104,105,106].

A positive effect, namely, the alleviation of cognitive deficit in in vivo models of schizophrenia, was also described resulting from activation of muscarinic acetylcholine receptors (specifically M_1_ and M_4_ receptors) present in the central nervous system [169,170]. Xanomeline, as an M_1_ and M_4_ receptor agonist, showed a beneficial effect in cognitive deficit in a clinical assay with 20 subjects [107]. Recently, it was shown that this drug induced beneficial effects in neuronal connectivity in animal models of schizophrenia [171].

The potentiation of cholinergic neurotransmission has not only been achieved by the direct activation of acetylcholine receptors. Another strategy to treat cognitive deficit in schizophrenia is the use of acetylcholinesterase inhibitors such as galantamine or donepezil, which prevent the degradation of acetylcholine in the synaptic cleft [172,173]. Galantamine has been assayed both in animal models of schizophrenia and in clinical trials in combination with memantine, an antagonist of NMDA glutamate receptors with procognitive effects [174]. Unfortunately, although the combination of both drugs seemed to be beneficial for cognitive improvement in animal models of schizophrenia [174], no robust benefits were observed in clinical trials with schizophrenic patients [108,175]. 

### 3.6. Potentiation of Glutamatergic Neurotransmission

As described above, schizophrenic patients with cognitive deficit show a reduction in NMDA receptor stimulation in DLPFC with imbalances in glutamate synthesis. Thus, several strategies have been proposed to tackle glutamatergic neurotransmission in order to relieve cognitive deficit in schizophrenic patients. One of them is the direct activation of NMDA receptors by employing CIQ isomers [(R) and (S)-(3-chlorophenyl) (6,7-dimethoxy-1-((4-methoxyphe-noxy)methyl)-3,4-dihydroisoquinolin-2(1H)-yl)meth-anone] [176], but because most animal models of schizophrenia employ NMDA antagonists to induce schizophrenia-like symptoms, it is not clear how translational those findings are. In animal models of schizophrenia it was observed that NMDA receptor antagonists such as memantine exert a beneficial effect for cognitive deficit [177], but this effect was not observed in clinical trials [109].

Another strategy to activate NMDA receptors is the enhancement of the function of co-agonists of the receptor such as glycine or serine. Inhibitors of glycine transporter-1 (GlyT1) have been widely studied as a treatment of cognitive deficit in schizophrenia because they elevate synaptic glycine levels [178]. However, studies assessing the clinical efficacy of the GlyT1 inhibitors have been contradictory: bitopertin failed to show a robust benefit in alleviating cognitive deficit [110], while BI 425809 showed a slight improvement in cognitive functions [111]. The different effect could be due to the small number of patients included in clinical trials, to the heterogeneity of clinical scales to assess cognitive deficit or to the short follow-up time in clinical trials [110,111,179]. On the other hand, cycloserine has been assayed as an activator of NMDA receptors, but the evidence of a positive effect on cognitive deficit in schizophrenia is not strong [180].

The activation of glutamate metabotropic receptors has been studied as a novel mechanism to improve cognitive deficit in schizophrenia because the activation of metabotropic receptors reduces the release of glutamate in cortical neurons, which, as exposed above, is paradoxically augmented in schizophrenia [181]. Results in mice are contradictory because a clear benefit was observed in some studies, while other studies failed to show a benefit of activating metabotropic receptors on cognitive deficit [182,183]. This lack of efficacy was also observed in clinical trials with LY2140023 [112]. Regardless, animal studies suggest that there are several strategies that appear to improve the effect of activating metabotropic receptors, such as administering them in adolescence, before the onset of schizophrenia symptoms [184,185]. Some studies have also shown a synergistic effect between the activation of glutamate metabotropic receptors and M_4_ acetylcholine receptors [186]. Shen et al. described that the activation of M_4_ receptors enhanced brain neuroplasticity [187], suggesting that the activation of metabotropic glutamate receptors requires an increase in neuronal plasticity to exert a beneficial effect on cognitive deficit.

The modulation of AMPA receptors has also been studied as an approach to enhance cognition in schizophrenic patients because the interplay between NMDA and AMPA receptors is critical for neuroplasticity [188]. The activation of AMPA receptors participates in cognitive processes such as learning and memory; however, AMPA agonists tend to induce AMPA receptor desensitization [189]. To avoid this effect, ampakines (allosteric potentiators of AMPA receptors) have been proposed to alleviate cognitive deficit, having been shown to improve cognitive functions in animal models of schizophrenia [190]. In clinical trials, the ampakine CX-516 has been shown to improve memory and attention in patients treated with clozapine [113], although it showed no clear beneficial effects in monotherapy [114].

### 3.7. Potentiation of Dopaminergic Neurotransmission

As described above, cognitive deficit in schizophrenia is related to a decrease in dopaminergic neurotransmission in DLPFC. Most dopamine receptors in DLPFC neurons are D_1_ receptors, which co-localize in dendritic spines with hyperpolarization-activated cyclic nucleotide-gated potassium channels (KCNQ1), whose dysfunction seem to be related to schizophrenia [191]. Because the relationship between dopaminergic neurotransmission and cognition follows an inverted U-shaped curve, both D_1_ agonists and antagonists have been assayed in animal models of schizophrenia as cognitive enhancers [192,193]. As dopaminergic neurotransmission refines the synaptic signals that reach the dendrites, the effect of dopaminergic drugs depends on the baseline levels of dopamine in the prefrontal cortex [194]. In the last few years, there has been a significant effort to develop D_1_ receptor positive allosteric modulators (PAMs) for cognitive deficit in schizophrenia (for a review see [195]). In phase 1 clinical trials, D_1_ receptor PAMs have been shown to be safe and tolerable [196]. However, one phase 2 clinical trial evaluating the procognitive efficacy of the D_1_ receptor PAM ASP4345 was stopped because the primary endpoint of the assay was not reached [197].

D_3_ receptor antagonism has been proposed as a mechanism for novel drugs against cognitive deficit in schizophrenia. The D_3_ receptor plays a crucial role in the regulation of dopamine release. Blockage of the D_3_ receptor enhances dopaminergic neurotransmission, with a beneficial effect on cognitive deficit [198]. On the other hand, the D_3_ receptor interacts with the nicotinic α_7_ receptor, boosting neuroplasticity (see [199] for a review). D_3_ and D_2_ receptor homology is very high, making it very difficult to develop drugs to selectively inhibit D_3_ receptors [200]. However, it has been proposed that the procognitive effects of several atypical antipsychotics may be due to their effects on D_3_ receptors (see [201] for a review).

Another strategy to increase the dopaminergic neurotransmission in DLPFC is the employment of the dopamine reuptake inhibitor modafinil. Modafinil has long been employed as a cognitive enhancer in both healthy individuals and in patients with neurodegenerative diseases and psychiatric pathologies [202]. Although it has been classified as a dopamine reuptake inhibitor, modafinil also modulates norepinephrine and serotonin transport, enhances glutamatergic neurotransmission and blocks GABAergic signaling [203]. It was suggested that modafinil improved cognitive functions in animal models of schizophrenia [204], but a recent systematic review by Ortiz-Orendain et al. concludes that the evidence for the efficacy of modafinil in alleviating cognitive deficit in schizophrenic patients is weak [116].

### 3.8. Antidepressant Drugs

Antidepressant drugs have been tested for the treatment of cognitive deficit in animal models of schizophrenia. Some of them, such as reboxetine or escitalopram, have been shown to be beneficial, especially in combination with different atypical antipsychotic drugs [205,206,207]. However, a meta-analysis highlighted that there was a high variability between assays, leading to the conclusion that there were no clinically relevant effects on cognition in schizophrenic patients [117]. These results take on new meaning in light of the recent study by Casarotto et al., in which the authors demonstrated that the mechanism of action of both typical and long-acting antidepressants is to enhance neuronal plasticity by binding to the TrkB BDNF receptor [151].

### 3.9. Inhibition of Phosphodiesterases

Phosphodiesterase inhibition is a promising mechanism of action for improving cognitive function in schizophrenia because cAMP and cGMP are second messengers of many receptors whose hypofunctions are involved in cognitive deficit in schizophrenia, such as dopamine or glutamate [208]. Preventing the degradation of cAMP and cGMP is expected to potentiate central neurotransmitters’ activity. From the eleven members of the phosphodiesterase family, phosphodiesterases 1, 2, 4, 5, 9, 10 and 11 are widely expressed in central nervous system (see [209] and [210] for a review). We will focus on phosphodiesterases 1, 4 and 10, as they are the most studied in schizophrenia-induced cognitive deficit.

Phosphodiesterase 1 (PDE1) is an enzyme related to oxidative stress that colocalizes with dopamine receptors [211]. Its inhibition in animal models of schizophrenia appeared to be useful in alleviating cognitive deficit as an adjuvant of antipsychotic therapies with anti-inflammatory effect [212,213].

Phosphodiesterase 4 (PDE4) is one of the most widely studied phosphodiesterases in schizophrenia. It interacts with DISC1 which, as exposed above, is involved in neurogenesis and whose malfunction is related to schizophrenia [214]. Roflumilast, an inhibitor of phosphodiesterase 4, and has been tested for the alleviation of cognitive deficit in schizophrenic patients, showing little improvement in either electrophysiological abnormalities or cognitive impairments elicited by schizophrenia [118,215]. Studies with more patients are needed in order to draw meaningful conclusions about the efficacy of roflumilast in schizophrenia-induced cognitive deficit.

Phosphodiesterase 10 (PDE10) has been described as a promising target for the treatment of many neurodegenerative and psychiatric diseases (see [216] for a review). Its blockage in animal models of schizophrenia using rodents and apes induces a beneficial effect in cognitive deficit [217,218]. TAK-063, a PDE10 inhibitor, has reached clinical trials, and although it was shown to be safe in phase 1 [219], it did not show a significant improvement in cognitive abilities in phase 2 studies with schizophrenic patients [119].

### 3.10. Steroids

Both cortical and sexual hormones are linked to schizophrenia. Chronic activation of the hypothalamic–pituitary–adrenal (HPA) axis has been suggested to play a role in the pathogenesis of schizophrenia [220]. As described above, both increased cortisol levels and blunted cortisol awakening response have been associated with a worse cognitive functioning in patients with schizophrenia. However, few assays have been performed to evaluate the efficacy of drugs counteracting the deleterious effect of cortical hormones on cognitive deficit in schizophrenic patients. The neuroprotective steroid dehydroepiandrosterone exerts a clear beneficial effect on cognitive function in animal models of schizophrenia [221], even though this effect is not always observed in clinical trials with schizophrenic patients, with only slight increases in attention and movement and visual skills [120,121]. It has been suggested that its beneficial effect may be achieved only in the early stages of the disease [222].

The role of sexual hormones, especially estrogens and progesterone, in the pathogenesis of schizophrenia is better known. Estrogens participate in neuronal development and regulation and exert a neuroprotective role [223]. It has been described that estrogen replacement therapy as an adjunctive to antipsychotics may exert a slight beneficial effect in cognitive functions in some schizophrenic patients [224]. However, estrogen replacement therapy increases the risk of uterine and breast cancer [225]. Raloxifene is a drug with estrogen-agonistic properties in the brain and estrogen-antagonistic properties in breast and uterus, reducing the risk of uterine and breast cancer. The effect of raloxifene in cognitive deficit is inconsistent, as it has been shown to have a beneficial effect in some trials [122,123], while no benefit was seen in others [124]. These differences could be explained by the heterogeneity of the patients included in each assay, because the effect of raloxifene may depend on the severity of schizophrenia and whether the patients are post- or premenopausal women.

Pregnenolone is a precursor of progesterone and other steroid hormones that has also been tested as a drug for cognitive impairment in schizophrenic patients due to its beneficial effect as a neurogenesis enhancer, anti-apoptotic agent, HPA axis modulator, enhancer of myelination and regulator of GABAergic and glutamatergic neurotransmission [226]. As with raloxifene, the effects of pregnenolone are inconsistent between different trials, with a benefit seen in several trials [121], while no benefit of pregnenolone was produced in others [125,126]. The reason for these differences between trials may be due to different baseline serum pregnenolone levels. High serum pregnenolone is correlated with lower improvements in cognitive function.

## 4. Conclusions and Future Perspectives

Despite the large amount of work done investigating the etiologies and treatments of cognitive deficit in schizophrenia, little progress has been made. There are several reasons for this slow development. One of them is the low translationality of the animal models of schizophrenia. Most rely on giving mice a drug such as MK-801 or scopolamine that induces schizophrenia-like symptoms. Schizophrenia is more complex than that and, as explained in the Introduction, neuroanatomical abnormalities, biochemical imbalances and genetic alterations concur in its etiology. To overcome this drawback, novel models of schizophrenia must be developed, including new animal models such as genetically modified mice [227] and in vitro models using immortalized neuronal cells relevant to schizophrenia [228], which could be helpful for early drug discovery.

Another reason for the high attrition rate is the holistic complexity of schizophrenia. Schizophrenia is a syndrome characterized by a series of symptoms and signs, and the alterations behind them vary between patients. In addition, even the physiological status of each patient influences these symptoms and signs. Therefore, a more personalized treatment should be prescribed for schizophrenic patients. The determination of biomarkers and the advances in pharmacogenomics could make it possible to identify the most appropriate treatments for each patient [229].

Lack of adherence to the treatment in patients with schizophrenia also hampers the discovery of novel efficient drugs for cognitive deficit in schizophrenia because it introduces biases in the measurement of drug efficacy. To overcome this drawback, a more personalized therapy with motivational interviews to emphasize the benefits of the treatment and to identify the problems associated with the therapy could improve patient adherence to treatment [230].

Several successful treatments used for cognitive deficit in schizophrenia exert their action by enhancing neuroplasticity. This suggests that the combination of those pharmacological treatments with non-pharmacological therapies such as cognitive training could represent an advancement in the treatment of cognitive deficit in schizophrenic patients.

## Figures and Tables

**Table 1:** Summary of the drugs mentioned in the review assayed in clinical trials with their mechanisms of action, the quality of the evidence and the observed effect.

Mechanism of Action	Drug	Quality of Evidence	Effect Observed	Reference
Antioxidant	PUFAs	Randomized trial	Counteraction of cortical thickness	[90]
	*N*-acetylcysteine	Randomized double-blind trial	Improvement in cognitive speed	[91]
	Minocycline	Randomized double-blind trials	Improvement in information processing speed	[92,93]
5-HT_1A_ agonism	Tandospirone	Randomized double-blind trial	Improvement in executive function and verbal memory	[94]
5-HT_3_ antagonism	Ondansetron	Meta-analysis	Slight improvement in some functions (visual memory)	[95]
5-HT_3_ antagonism + α_7_ nicotinic agonism	Tropisetron	Randomized double-blind trial	Improvement in memory	[96]
5-HT_6_ antagonism	AVN-211	Randomized double-blind trial	Contradictory effects on cognitive domains	[97,98]
Non-selective GABA receptor agonists	Benzodiazepines	Observational study	Attention and working memory impairment	[99]
GABA prodrug	BL-1020	Randomized double-blind trial (Phase 2)	Improvement in a composite score	[100]
		Randomized double-blind trial (Phase 2_b_-3)	No benefits	[101]
H_3_ receptor antagonist	ABT-288	Randomized double-blind trial (Phase 2)	No benefits	[102]
α_7_ nicotinic receptor agonist	Varenicline	Meta-analysis	No benefits	[103]
	Encenicline	Randomized double-blind trials (Phase 3)	No benefits	[104]
	Nelonicline	Randomized double-blind trial (Phase 2_b_)	No benefits	[105]
	Bradanicline	Randomized double-blind trial (Phase 2)	No benefits	[106]
M_1_ and M_4_ muscarinic receptors agonist	Xanomeline	Randomized double-blind trial (pilot study)	Slight improvement in verbal learning and memory function	[107]
Acetylcholinesterase inhibitor	Galantamine	Meta-analysis	No clear improvement in memory, executive functioning, attention or reaction time	[108]
NMDA receptor antagonist	Memantine	Systematic review of open label or double-blind trials	No benefits	[109]
Inhibitors of glycine transporters	Bitopertin	Randomized double-blind trial	No benefits	[110]
	BI425809	Randomized double-blind trial (Phase 2)	Slight increase in a composite score	[111]
Activator of glutamate metabotropic receptors	LY2140023	Randomized double-blind trial	No benefit	[112]
Allosteric activator of AMPA receptors	CX-516	Randomized single-blind trial	Improvement in attention and memory (combined with clozapine)	[113]
		Randomized double-blind trial (4 patients)	No benefit	[114]
D_1_ receptor positive allosteric modulator (PAM)	ASP4345	Randomized double-blind trial (Phase 2)	No benefit	[115]
Dopamine reuptake inhibitor	Modafinil	Systematic review	No benefit	[116]
Antidepressants	Antidepressants belonging to various classes	Meta-analysis	No clear benefits of the combination of antidepressants and antipsychotics	[117]
Phosphodiesterase 4 inhibitor	Roflumilast	Randomized double-blind trial	Verbal memory improvement	[118]
Phosphodiesterase 10 inhibitor	TAK-063	Randomized double-blind trial (Phase 2)	No benefit	[119]
Neuroprotective steroid	Dehydroepiandrosterone	Randomized double-blind trial	Slight improvement in attention and visual and movement skills	[120]
Neuroprotective steroid	Dehydroepiandrosterone	Randomized double-blind trial	No benefit	[121]
Estrogen agonist in brain	Raloxifene	Randomized double-blind trials	Improvement in verbal memory and other cognitive domains	[122,123]
	Raloxifene	Randomized double-blind trial	No benefit	[124]
Progesterone precursor	Pregnenolone	Randomized double-blind trial	Improvement in memory and working attention	[121]
		Randomized double-blind trials	No benefit	[125,126]

## Data Availability

Not applicable.

## References

[1] Mueser K.T., McGurk S.R. (2004). Schizophrenia. Lancet.

[2] Carrà G., Crocamo C., Angermeyer M., Brugha T., Toumi M., Bebbington P. (2019). Positive and negative symptoms in schizophrenia: A longitudinal analysis using latent variable structural equation modelling. Schizophr. Res..

[3] Chang C.-Y., Luo D.-Z., Pei J.-C., Kuo M.-C., Hsieh Y.-C., Lai W.-S. (2021). Not Just a Bystander: The Emerging Role of Astrocytes and Research Tools in Studying Cognitive Dysfunctions in Schizophrenia. Int. J. Mol. Sci..

[4] Dollfus S., Lyne J. (2017). Negative symptoms: History of the concept and their position in diagnosis of schizophrenia. Schizophr. Res..

[5] Bora E. (2019). Peripheral inflammatory and neurotrophic biomarkers of cognitive impairment in schizophrenia: A meta-Analysis. Psychol. Med..

[6] Mesholam-Gately R.I., Giuliano A.J., Goff K.P., Faraone S.V., Seidman L.J. (2009). Neurocognition in First-Episode Schizophrenia: A Meta-Analytic Review. Neuropsychology.

[7] Xu M.-Y., Wong A.H.C. (2018). GABAergic inhibitory neurons as therapeutic targets for cognitive impairment in schizophrenia. Acta Pharmacol. Sin..

[8] Bezdicek O., Michalec J., Kališová L., Kufa T., Děchtěrenko F., Chlebovcová M., Havlík F., Green M.F., Nuechterlein K.H. (2020). Profile of cognitive deficits in schizophrenia and factor structure of the Czech MATRICS Consensus Cognitive Battery. Schizophr. Res..

[9] Hoff A.L., Riordan H., O’Donnell D.W., Morris L., DeLisi L.E. (1992). Neuropsychological functioning of first-episode schizophreniform patients. Am. J. Psychiatry.

[10] Xie T., Zhang X., Tang X., Zhang H., Yu M., Gong G., Wang X., Evans A., Zhang Z., He Y. (2019). Mapping convergent and divergent cortical thinning patterns in patients with deficit and nondeficit schizophrenia. Schizophr. Bull..

[11] Haijma S.V., Van Haren N., Cahn W., Koolschijn P.C.M.P., Hulshoff Pol H.E., Kahn R.S. (2013). Brain volumes in schizophrenia: A meta-analysis in over 18 000 subjects. Schizophr. Bull..

[12] Planchuelo-Gómez Á., Lubeiro A., Núñez-Novo P., Gomez-Pilar J., de Luis-García R., del Valle P., Martín-Santiago Ó., Pérez-Escudero A., Molina V. (2020). Identificacion of MRI-based psychosis subtypes: Replication and refinement. Prog. Neuro-Psychopharmacol. Biol. Psychiatry.

[13] Gould I.C., Shepherd A.M., Laurens K.R., Cairns M.J., Carr V.J., Green M.J. (2014). Multivariate neuroanatomical classification of cognitive subtypes in schizophrenia: A support vector machine learning approach. NeuroImage Clin..

[14] Brugger S.P., Howes O.D. (2017). Heterogeneity and Homogeneity of Regional Brain Structure in Schizophrenia: A Meta-analysis. JAMA Psychiatry.

[15] Alústiza I., Radua J., Albajes-Eizagirre A., Domínguez M., Aubá E., Ortuño F. (2016). Meta-Analysis of Functional Neuroimaging and Cognitive Control Studies in Schizophrenia: Preliminary Elucidation of a Core Dysfunctional Timing Network. Front. Psychol..

[16] Elsworth J.D., Morrow B.A., Hajszan T., Leranth C., Roth R.H. (2011). Phencyclidine-induced Loss of Asymmetric Spine Synapses in Rodent Prefrontal Cortex is Reversed by Acute and Chronic Treatment with Olanzapine. Neuropsychopharmacology.

[17] Ji J.L., Diehl C., Schleifer C., Tamminga C.A., Keshavan M.S., Sweeney J.A., Clementz B.A., Hill S.K., Pearlson G., Yang G. (2019). Schizophrenia Exhibits Bi-directional Brain-Wide Alterations in Cortico-Striato-Cerebellar Circuits. Cereb. Cortex.

[18] Huang M.L., Khoh T.T., Lu S.J., Pan F., Chen J.K., Hu J.B., Hu S.H., Xu W.J., Zhou W.H., Wei N. (2017). Relationships between dorsolateral prefrontal cortex metabolic change and cognitive impairment in first-episode neuroleptic-naive schizophrenia patients. Medicine.

[19] Ellegood J., Markx S., Lerch J.P., Steadman P.E., Genç C., Provenzano F., Kushner S.A., Henkelman R.M., Karayiorgou M., Gogos J.A. (2014). Neuroanatomical phenotypes in a mouse model of the 22q11.2 microdeletion. Mol. Psychiatry.

[20] Schretlen D.J., Vannorsdall T.D., Winicki J.M., Mushtaq Y., Hikida T., Sawa A., Yolken R.H., Dickerson F.B., Cascella N.G. (2010). Neuroanatomic and cognitive abnormalities related to herpes simplex virus type 1 in schizophrenia. Schizophr. Res..

[21] Weinberger D.R. (2017). The neurodevelopmental origins of schizophrenia in the penumbra of genomic medicine. World Psychiatry.

[22] Murray R.M., Lewis S.W. (1987). Is schizophrenia a neurodevelopmental disorder?. BMJ.

[23] Tripathi A., Kar S.K., Shukla R. (2018). Cognitive deficits in schizophrenia: Understanding the biological correlates and remediation strategies. Clin. Psychopharmacol. Neurosci..

[24] Snyder M.A., Gao W.J. (2013). NMDA hypofunction as a convergence point for progression and symptoms of schizophrenia. Front. Cell. Neurosci..

[25] Fathian F., Loberg E.M., Gjestad R., Steen V.M., Kroken R.A., Jorgensen H.A., Johnsen E. (2019). Associations between C-reactive protein levels and cognition during the first 6 months after acute psychosis. Acta Neuropsychiatr..

[26] Fillman S.G., Weickert T.W., Lenroot R.K., Catts S.V., Bruggemann J.M., Catts V.S., Weickert C.S. (2016). Elevated peripheral cytokines characterize a subgroup of people with schizophrenia displaying poor verbal fluency and reduced Broca’s area volume. Mol. Psychiatry.

[27] Ribeiro-Santos R., de Campos-Carli S.M., Ferretjans R., Teixeira-Carvalho A., Martins-Filho O.A., Teixeira A.L., Salgado J.V. (2020). The association of cognitive performance and IL-6 levels in schizophrenia is influenced by age and antipsychotic treatment. Nord. J. Psychiatry.

[28] Baune B.T., Ponath G., Rothermundt M., Riess O., Funke H., Berger K. (2008). Association between genetic variants of IL-1β, IL-6 and TNF-α cytokines and cognitive performance in the elderly general population of the MEMO-study. Psychoneuroendocrinology.

[29] Lupien S.J., Juster R.P., Raymond C., Marin M.F. (2018). The effects of chronic stress on the human brain: From neurotoxicity, to vulnerability, to opportunity. Front. Neuroendocrinol..

[30] Mondelli V., Cattaneo A., Murri M.B., Papadopoulos A.S., Aitchison K.J. (2011). Stress and inflammation reduce BDNF expression in first- episode psychosis: A pathway to smaller hippocampal volume. J. Clin. Psychiatry.

[31] Mondelli V., Pariante C.M., Navari S., Aas M., D’Albenzio A., Di Forti M., Handley R., Hepgul N., Marques T.R., Taylor H. (2010). Higher cortisol levels are associated with smaller left hippocampal volume in first-episode psychosis. Schizophr. Res..

[32] Aas M., Dazzan P., Mondelli V., Toulopoulou T., Reichenberg A., Di Forti M., Fisher H.L., Handley R., Hepgul N., Marques T. (2011). Abnormal cortisol awakening response predicts worse cognitive function in patients with first-episode psychosis. Psychol. Med..

[33] Havelka D., Prikrylova-Kucerova H., Prikryl R., Ceskova E. (2016). Cognitive impairment and cortisol levels in first-episode schizophrenia patients. Stress.

[34] Labad J. (2019). The role of cortisol and prolactin in the pathogenesis and clinical expression of psychotic disorders. Psychoneuroendocrinology.

[35] Goodnick P.J., Santana O., Rodriguez L. (2002). Antipsychotics: Impact on prolactin levels. Expert Opin. Pharmacother..

[36] Montalvo I., Gutiérrez-Zotes A., Creus M., Monseny R., Ortega L., Franch J., Lawrie S.M., Reynolds R.M., Vilella E., Labad J. (2014). Increased prolactin levels are associated with impaired processing speed in subjects with early psychosis. PLoS ONE.

[37] Yao S., Song J., Gao J., Lin P., Yang M., Zahid K.R., Yan Y., Cao C., Ma P., Zhang H. (2018). Cognitive function and serum hormone levels are associated with gray matter volume decline in female patients with prolactinomas. Front. Neurol..

[38] García-Rizo C., Vázquez-Bourgon J., Labad J., Ortiz García de la Foz V., Gómez-Revuelta M., Juncal Ruiz M., Crespo-Facorro B. (2021). Prolactin, metabolic and immune parameters in naïve subjects with a first episode of psychosis. Prog. Neuro-Psychopharmacol. Biol. Psychiatry.

[39] Hori H., Yoshimura R., Katsuki A., Atake K., Igata R., Konishi Y., Nakamura J. (2017). Relationships between Serum Brain-Derived Neurotrophic Factor, Plasma Catecholamine Metabolites, Cytokines, Cognitive Function and Clinical Symptoms in Japanese Patients with Chronic Schizophrenia Treated with Atypical Antipsychotic Monotherapy. World J. Biol. Psychiatry.

[40] Yang Y., Liu Y., Wang G., Hei G., Wang X., Li R., Li L., Wu R., Zhao J. (2019). Brain-derived neurotrophic factor is associated with cognitive impairments in first-episode and chronic schizophrenia. Psychiatry Res..

[41] Vasconcelos G.S., Ximenes N.C., de Sousa C.N.S., Oliveira T., de Lima L.L.L., de Lucena D.F., Gama C.S., Macêdo D., Vasconcelos S.M.M. (2015). Alpha-lipoic acid alone and combined with clozapine reverses schizophrenia-like symptoms induced by ketamine in mice: Participation of antioxidant, nitrergic and neurotrophic mechanisms. Schizophr. Res..

[42] Ertuĝrul A., Özdemir H., Vural A., Dalkara T., Meltzer H.Y., Saka E. (2011). The influence of N-desmethylclozapine and clozapine on recognition memory and BDNF expression in hippocampus. Brain Res. Bull..

[43] Zhang X.Y., Chen D.C., Xiu M.H., Haile C.N., Luo X., Xu K., Zhang H.P., Zuo L., Zhang Z., Zhang X. (2012). Cognitive and serum BDNF correlates of BDNF Val66Met gene polymorphism in patients with schizophrenia and normal controls. Hum. Genet..

[44] Ho B.-C., Milev P., O’Leary D.S., Librant A., Andreasen N.C., Wassink T.H. (2006). Cognitive and Magnetic Resonance Imaging Brain Morphometric Correlates of Brain-Derived Neurotrophic Factor Val66Met Gene Polymorphism in Patients with Schizophrenia and Healthy Volunteers. Arch. Gen. Psychiatry.

[45] Huang M., Panos J.J., Kwon S., Oyamada Y., Rajagopal L., Meltzer H.Y. (2014). Comparative effect of lurasidone and blonanserin on cortical glutamate, dopamine, and acetylcholine efflux: Role of relative serotonin (5-HT) 2A and da D2 antagonism and 5-HT1A partial agonism. J. Neurochem..

[46] Schoonover K.E., Dienel S.J., Lewis D.A. (2020). Prefrontal cortical alterations of glutamate and GABA neurotransmission in schizophrenia: Insights for rational biomarker development. Biomark. Neuropsychiatry.

[47] Fang X., Wang Y., Cheng L., Zhang Y., Zhou Y., Wu S., Huang H., Zou J., Chen C., Chen J. (2018). Prefrontal dysconnectivity links to working memory deficit in first-episode schizophrenia. Brain Imaging Behav..

[48] Chiu P.W., Lui S.S.Y., Hung K.S.Y., Chan R.C.K., Chan Q., Sham P.C., Cheung E.F.C., Mak H.K.F. (2018). In vivo gamma-aminobutyric acid and glutamate levels in people with first-episode schizophrenia: A proton magnetic resonance spectroscopy study. Schizophr. Res..

[49] Cho R.Y., Konecky R.O., Carter C.S. (2006). Impairments in frontal cortical γ synchrony and cognitive control in schizophrenia. Proc. Natl. Acad. Sci. USA.

[50] Weickert C.S., Fung S.J., Catts V.S., Schofield P.R., Allen K.M., Moore L.T., Newell K.A., Pellen D., Huang X.F., Catts S.V. (2013). Molecular evidence of N-methyl-D-aspartate receptor hypofunction in schizophrenia. Mol. Psychiatry.

[51] Billard J.-M. (2018). Changes in Serine Racemase-Dependent Modulation of NMDA Receptor: Impact on Physiological and Pathological Brain Aging. Front. Mol. Biosci..

[52] Sathyasaikumar K.V., Stachowski E.K., Wonodi I., Roberts R.C., Rassoulpour A., McMahon R.P., Schwarcz R. (2011). Impaired Kynurenine Pathway Metabolism in The Prefrontal Cortex of Individuals with Schizophrenia. Schizophr. Bull..

[53] Kindler J., Lim C.K., Weickert C.S., Boerrigter D., Galletly C., Liu D., Jacobs K.R., Balzan R., Bruggemann J., O’Donnell M. (2020). Dysregulation of kynurenine metabolism is related to proinflammatory cytokines, attention, and prefrontal cortex volume in schizophrenia. Mol. Psychiatry.

[54] Jones C.A., Watson D.J.G., Fone K.C.F. (2011). Animal models of schizophrenia. Br. J. Pharmacol..

[55] Kaminski J., Mascarell-Maricic L., Fukuda Y., Katthagen T., Heinz A., Schlagenhauf F. (2021). Glutamate in the Dorsolateral Prefrontal Cortex in Patients with Schizophrenia: A Meta-analysis of 1H-Magnetic Resonance Spectroscopy Studies. Biol. Psychiatry.

[56] Gluck M.R., Thomas R.G., Davis K.L., Haroutunian V. (2002). Implications for Altered Glutamate and GABA Metabolism in the Dorsolateral Prefrontal Cortex of Aged Schizophrenic Patients. Am. J. Psychiatry.

[57] Volk D.W., Eggan S.M., Lewis D.A. (2010). Alterations in Metabotropic Glutamate Receptor 1α and Regulator of G Protein Signaling 4 in the Prefrontal Cortex in Schizophrenia. Am. J. Psychiatry.

[58] Upreti C., Zhang X., Alford S., Stanton P.K. (2013). Role of presynaptic metabotropic glutamate receptors in the induction of long-term synaptic plasticity of vesicular release. Neuropharmacology.

[59] Whittington M.A., Cunningham M.O., LeBeau F.E.N., Racca C., Traub R.D. (2011). Multiple origins of the cortical gamma rhythm. Dev. Neurobiol..

[60] Volk D.W., Austin M.C., Pierri J.N., Sampson A.R., Lewis D.A. (2000). Decreased Glutamic Acid Decarboxylase67 Messenger RNA Expression in a Subset of Prefrontal Cortical γ-Aminobutyric Acid Neurons in Subjects with Schizophrenia. Arch. Gen. Psychiatry.

[61] Enwright J.F., Sanapala S., Foglio A., Berry R., Fish K.N., Lewis D.A. (2016). Reduced Labeling of Parvalbumin Neurons and Perineuronal Nets in the Dorsolateral Prefrontal Cortex of Subjects with Schizophrenia. Neuropsychopharmacology.

[62] Liu Y., Yang X.J., Xia H., Tang C.-M., Yang K. (2019). GABA releases from parvalbumin-expressing and unspecific GABAergic neurons onto CA1 pyramidal cells are differentially modulated by presynaptic GABAB receptors in mouse hippocampus. Biochem. Biophys. Res. Commun..

[63] Lewis D.A. (2011). The chandelier neuron in schizophrenia. Dev. Neurobiol..

[64] Morris H.M., Hashimoto T., Lewis D.A. (2008). Alterations in Somatostatin mRNA Expression in the Dorsolateral Prefrontal Cortex of Subjects with Schizophrenia or Schizoaffective Disorder. Cereb. Cortex.

[65] Avram M., Brandl F., Cabello J., Leucht C., Scherr M., Mustafa M., Leucht S., Ziegler S., Sorg C. (2019). Reduced striatal dopamine synthesis capacity in patients with schizophrenia during remission of positive symptoms. Brain.

[66] Avram M., Brandl F., Knolle F., Cabello J., Leucht C., Scherr M., Mustafa M., Koutsouleris N., Leucht S., Ziegler S. (2020). Aberrant striatal dopamine links topographically with cortico-thalamic dysconnectivity in schizophrenia. Brain.

[67] Koola M.M., Looney S.W., Hong H., Pillai A., Hou W. (2020). Meta-analysis of randomized controlled trials of galantamine in schizophrenia: Significant cognitive enhancement. Psychiatry Res..

[68] Bakker G., Vingerhoets C., Bloemen O.J.N., Sahakian B.J., Booij J., Caan M.W.A., van Amelsvoort T.A.M.J. (2020). The muscarinic M1 receptor modulates associative learning and memory in psychotic disorders. NeuroImage Clin..

[69] Jin C.Y., Anichtchik O., Panula P. (2009). Altered histamine H 3 receptor radioligand binding in post-mortem brain samples from subjects with psychiatric diseases. Br. J. Pharmacol..

[70] Zai G., Robbins T.W., Sahakian B.J., Kennedy J.L. (2017). A review of molecular genetic studies of neurocognitive deficits in schizophrenia. Neurosci. Biobehav. Rev..

[71] Apud J.A., Weinberger D.R. (2007). Treatment of cognitive deficits associated with schizophrenia: Potential role of catechol-O-methyltransferase inhibitors. CNS Drugs.

[72] Burton C.Z., Vella L., Kelsoe J.R., Bilder R.M., Twamley E.W. (2015). Catechol-O-methyltransferase genotype and response to Compensatory Cognitive Training in outpatients with schizophrenia. Psychiatr. Genet..

[73] Malhotra A.K., Kestler L.J., Mazzanti C., Bates J.A., Goldberg T., Goldman D. (2002). A Functional Polymorphism in the COMT Gene and Performance on a Test of Prefrontal Cognition. Am. J. Psychiatry.

[74] Pinheiro A.P., Keefe R.S.E., Skelly T., Olarte M., Leviel K., Lange L.A., Lange E.M., Stroup T.S., Lieberman J., Sullivan P.F. (2007). AKT1 and neurocognition in schizophrenia. Aust. N. Z. J. Psychiatry.

[75] Huang C.H., Pei J.C., Luo D.Z., Chen C., Chen Y.W., Lai W.S. (2015). Investigation of gene effects and epistatic interactions between Akt1 and neuregulin 1 in the regulation of behavioral phenotypes and social functions in genetic mouse models of schizophrenia. Front. Behav. Neurosci..

[76] Tang T.T.T., Yang F., Chen B.S., Lu Y., Ji Y., Roche K.W., Lu B. (2009). Dysbindin regulates hippocampal LTP by controlling NMDA receptor surface expression. Proc. Natl. Acad. Sci. USA.

[77] Al-Shammari A.R., Bhardwaj S.K., Musaelyan K., Srivastava L.K., Szele F.G. (2018). Schizophrenia-related dysbindin-1 gene is required for innate immune response and homeostasis in the developing subventricular zone. npj Schizophr..

[78] Yang Y., Zhang L., Guo D., Zhang L., Yu H., Liu Q., Su X., Shao M., Song M., Zhang Y. (2020). Association of DTNBP1 With Schizophrenia: Findings from Two Independent Samples of Han Chinese Population. Front. Psychiatry.

[79] Zhang J.P., Burdick K.E., Lencz T., Malhotra A.K. (2010). Meta-analysis of genetic variation in DTNBP1 and general cognitive ability. Biol. Psychiatry.

[80] Tropea D., Hardingham N., Millar K., Fox K. (2018). Mechanisms underlying the role of DISC1 in synaptic plasticity. J. Physiol..

[81] Teng S., Thomson P.A., McCarthy S., Kramer M., Muller S., Lihm J., Morris S., Soares D.C., Hennah W., Harris S. (2018). Rare disruptive variants in the DISC1 Interactome and Regulome: Association with cognitive ability and schizophrenia. Mol. Psychiatry.

[82] Meltzer H.Y. (2013). Update on typical and atypical antipsychotic drugs. Annu. Rev. Med..

[83] Meltzer H.Y., Matsubara S., Lee J.C. (1989). Classification of typical and atypical antipsychotic drugs on the basis of dopamine D-1, D-2 and serotonin2 pKi values. J. Pharmacol. Exp. Ther..

[84] Gray J.A., Roth B.L. (2007). Molecular Targets for Treating Cognitive Dysfunction in Schizophrenia. Schizophr. Bull..

[85] Nielsen R.E., Levander S., Kjaersdam Telléus G., Jensen S.O.W., Østergaard Christensen T., Leucht S. (2015). Second-generation antipsychotic effect on cognition in patients with schizophrenia—A meta-analysis of randomized clinical trials. Acta Psychiatr. Scand..

[86] Désaméricq G., Schurhoff F., Meary A., Szöke A., Macquin-Mavier I., Bachoud-Lévi A.C., Maison P. (2014). Long-term neurocognitive effects of antipsychotics in schizophrenia: A network meta-analysis. Eur. J. Clin. Pharmacol..

[87] Baldez D.P., Biazus T.B., Rabelo-da-Ponte F.D., Nogaro G.P., Martins D.S., Kunz M., Czepielewski L.S. (2021). The effect of antipsychotics on the cognitive performance of individuals with psychotic disorders: Network meta-analyses of randomized controlled trials. Neurosci. Biobehav. Rev..

[88] De la Fuente Revenga M., Ibi D., Saunders J.M., Cuddy T., Ijaz M.K., Toneatti R., Kurita M., Holloway T., Shen L., Seto J. (2018). HDAC2-dependent Antipsychotic-like Effects of Chronic Treatment with the HDAC Inhibitor SAHA in Mice. Neuroscience.

[89] Ibi D., de la Fuente Revenga M., Kezunovic N., Muguruza C., Saunders J.M., Gaitonde S.A., Moreno J.L., Ijaz M.K., Santosh V., Kozlenkov A. (2017). Antipsychotic-induced Hdac2 transcription via NF-κB leads to synaptic and cognitive side effects. Nat. Neurosci..

[90] Pawełczyk T., Piątkowska-Janko E., Bogorodzki P., Gębski P., Grancow-Grabka M., Trafalska E., Żurner N., Pawełczyk A. (2018). Omega-3 fatty acid supplementation may prevent loss of gray matter thickness in the left parieto-occipital cortex in first episode schizophrenia: A secondary outcome analysis of the OFFER randomized controlled study. Schizophr. Res..

[91] Conus P., Seidman L.J., Fournier M., Xin L., Cleusix M., Baumann P.S., Ferrari C., Cousins A., Alameda L., Gholam-Rezaee M. (2018). N-acetylcysteine in a double-blind randomized placebo-controlled trial: Toward biomarker-guided treatment in early psychosis. Schizophr. Bull..

[92] Zhang L., Zheng H., Wu R., Kosten T.R., Zhang X.-Y., Zhao J. (2019). The effect of minocycline on amelioration of cognitive deficits and pro-inflammatory cytokines levels in patients with schizophrenia. Schizophr. Res..

[93] Liu F., Guo X., Wu R., Ou J., Zheng Y., Zhang B., Xie L., Zhang L., Yang L., Yang S. (2014). Minocycline supplementation for treatment of negative symptoms in early-phase schizophrenia: A double blind, randomized, controlled trial. Schizophr. Res..

[94] Sumiyoshi T., Matsui M., Yamashita I., Nohara S., Uehara T., Kurachi M., Meltzer H.Y. (2000). Effect of adjunctive treatment with serotonin-1A agonist tandospirone on memory functions in schizophrenia. J. Clin. Psychopharmacol..

[95] Zheng W., Cai D.-B., Zhang Q.-E., He J., Zhong L.-Y., Sim K., Ungvari G.S., Ning Y.-P., Xiang Y.-T. (2019). Adjunctive ondansetron for schizophrenia: A systematic review and meta-analysis of randomized controlled trials. J. Psychiatr. Res..

[96] Xia L., Liu L., Hong X., Wang D., Wei G., Wang J., Zhou H., Xu H., Tian Y., Dai Q. (2020). One-day tropisetron treatment improves cognitive deficits and P50 inhibition deficits in schizophrenia. Neuropsychopharmacology.

[97] Morozova M.A., Lepilkina T.A., Rupchev G.E., Beniashvily A.G., Burminskiy D.S., Potanin S.S., Bondarenko E.V., Kazey V.I., Lavrovsky Y., Ivachtchenko A.V. (2014). Add-on clinical effects of selective antagonist of 5HT6 receptors AVN-211 (CD-008-0173) in patients with schizophrenia stabilized on antipsychotic treatment: Pilot study. CNS Spectr..

[98] Morozova M., Burminskiy D., Rupchev G., Lepilkina T., Potanin S., Beniashvili A., Lavrovsky Y., Vostokova N., Ivaschenko A. (2017). 5-HT6 Receptor Antagonist as an Adjunct Treatment Targeting Residual Symptoms in Patients with Schizophrenia. J. Clin. Psychopharmacol..

[99] Fond G., Berna F., Boyer L., Godin O., Brunel L., Andrianarisoa M., Aouizerate B., Capdevielle D., Chereau I., Danion J.M. (2018). Benzodiazepine long-term administration is associated with impaired attention/working memory in schizophrenia: Results from the national multicentre FACE-SZ data set. Eur. Arch. Psychiatry Clin. Neurosci..

[100] Geffen Y., Keefe R., Rabinowitz J., Anand R., Davidson M. (2012). BL-1020, a New γ-Aminobutyric Acid–Enhanced Antipsychotic. J. Clin. Psychiatry.

[101] Phase IIb-III Study of BL-1020 Small Molecule for Schizophrenia (CLARITY). https://clinicaltrials.gov/ct2/show/results/NCT01363349?term=bl-1020&draw=2&rank=3.

[102] Haig G.M., Bain E., Robieson W., Othman A.A., Baker J., Lenz R.A. (2014). A randomized trial of the efficacy and safety of the H3 antagonist ABT-288 in cognitive impairment associated with schizophrenia. Schizophr. Bull..

[103] Tanzer T., Shah S., Benson C., De Monte V., Gore-Jones V., Rossell S.L., Dark F., Kisely S., Siskind D., Melo C.D. (2020). Varenicline for cognitive impairment in people with schizophrenia: Systematic review and meta-analysis. Psychopharmacology.

[104] Brannan S. (2019). Two global phase III trials of encenicline for cognitive impairment in chronic schizophrenia patients: Red flags and lessons learned. Schizophr. Bull..

[105] Haig G.M., Wang D., Zhao J., Othman A.A., Bain E.E. (2018). Efficacy and Safety of the α7-Nicotinic Acetylcholine Receptor Agonist ABT-126 in the Treatment of Cognitive Impairment Associated with Schizophrenia. J. Clin. Psychiatry.

[106] Walling D., Marder S.R., Kane J., Fleischhacker W.W., Keefe R.S.E., Hosford D.A., Dvergsten C., Segreti A.C., Beaver J.S., Toler S.M. (2016). Phase 2 Trial of an Alpha-7 Nicotinic Receptor Agonist (TC-5619) in Negative and Cognitive Symptoms of Schizophrenia. Schizophr. Bull..

[107] Shekhar A., Potter W.Z., Lightfoot J., Lienemann J., Dubé S., Mallinckrodt C., Bymaster F.P., McKinzie D.L., Felder C.C. (2008). Selective muscarinic receptor agonist xanomeline as a novel treatment approach for schizophrenia. Am. J. Psychiatry.

[108] Singh J., Kour K., Jayaram M.B. (2012). Acetylcholinesterase inhibitors for schizophrenia. Cochrane Database Syst. Rev..

[109] Di Iorio G., Baroni G., Lorusso M., Montemitro C., Spano M.C., di Giannantonio M. (2017). Efficacy of Memantine in Schizophrenic Patients: A Systematic Review. J. Amino Acids.

[110] Kantrowitz J.T., Nolan K.A., Epstein M.L., Lehrfeld N., Shope C., Petkova E., Javitt D.C. (2017). Neurophysiological Effects of Bitopertin in Schizophrenia. J. Clin. Psychopharmacol..

[111] Fleischhacker W.W., Podhorna J., Gröschl M., Hake S., Zhao Y., Huang S., Keefe R.S.E., Desch M., Brenner R., Walling D.P. (2021). Efficacy and safety of the novel glycine transporter inhibitor BI 425809 once daily in patients with schizophrenia: A double-blind, randomised, placebo-controlled phase 2 study. Lancet Psychiatry.

[112] Downing A.M., Kinon B.J., Millen B.A., Zhang L., Liu L., Morozova M.A., Brenner R., Rayle T.J., Nisenbaum L., Zhao F. (2014). A double-blind, placebo-controlled comparator study of LY2140023 monohydrate in patients with schizophrenia. BMC Psychiatry.

[113] Goff D.C., Leahy L., Berman I., Posever T., Herz L., Leon A.C., Johnson S.A., Lynch G. (2001). A Placebo-Controlled Pilot Study of the Ampakine CX516 Added to Clozapine in Schizophrenia. J. Clin. Psychopharmacol..

[114] Marenco S., Egan M.F., Goldberg T.E., Knable M.B., McClure R.K., Winterer G., Weinberger D.R. (2002). Preliminary experience with an ampakine (CX516) as a single agent for the treatment of schizophrenia: A case series. Schizophr. Res..

[115] Astellas Pharma Global Development A Phase 2a, Randomized, Double-Blind, Placebo-Controlled, Parallel-group Study to Assess the Safety and Efficacy of ASP4345 as Add-on Treatment for Cognitive Impairment in Subjects with Schizophrenia on Stable Doses of Antipsychotic Medication. https://astellasclinicalstudyresults.com/study.aspx?ID=404.

[116] Ortiz-Orendain J., Covarrubias-Castillo S.A., Vazquez-Alvarez A.O., Castiello-de Obeso S., Arias Quiñones G.E., Seegers M., Colunga-Lozano L.E. (2019). Modafinil for people with schizophrenia or related disorders. Cochrane Database Syst. Rev..

[117] Vernon J.A., Grudnikoff E., Seidman A.J., Frazier T.W., Vemulapalli M.S., Pareek P., Goldberg T.E., Kane J.M., Correll C.U. (2014). Antidepressants for cognitive impairment in schizophrenia—A systematic review and meta-analysis. Schizophr. Res..

[118] Gilleen J., Farah Y., Davison C., Kerins S., Valdearenas L., Uz T., Lahu G., Tsai M., Ogrinc F., Reichenberg A. (2018). An experimental medicine study of the phosphodiesterase-4 inhibitor, roflumilast, on working memory-related brain activity and episodic memory in schizophrenia patients. Psychopharmacology.

[119] Macek T.A., McCue M., Dong X., Hanson E., Goldsmith P., Affinito J., Mahableshwarkar A.R. (2019). A phase 2, randomized, placebo-controlled study of the efficacy and safety of TAK-063 in subjects with an acute exacerbation of schizophrenia. Schizophr. Res..

[120] Ritsner M.S., Gibel A., Ratner Y., Tsinovoy G., Strous R.D. (2006). Improvement of Sustained Attention and Visual and Movement Skills, but Not Clinical Symptoms, after Dehydroepiandrosterone Augmentation in Schizophrenia. J. Clin. Psychopharmacol..

[121] Ritsner M.S., Gibel A., Shleifer T., Boguslavsky I., Zayed A., Maayan R., Weizman A., Lerner V. (2010). Pregnenolone and Dehydroepiandrosterone as an Adjunctive Treatment in Schizophrenia and Schizoaffective Disorder. J. Clin. Psychiatry.

[122] Weickert T.W., Weinberg D., Lenroot R., Catts S.V., Wells R., Vercammen A., O’Donnell M., Galletly C., Liu D., Balzan R. (2015). Adjunctive raloxifene treatment improves attention and memory in men and women with schizophrenia. Mol. Psychiatry.

[123] Gurvich C., Hudaib A., Gavrilidis E., Worsley R., Thomas N., Kulkarni J. (2019). Raloxifene as a treatment for cognition in women with schizophrenia: The influence of menopause status. Psychoneuroendocrinology.

[124] Weiser M., Levi L., Burshtein S., Hagin M., Matei V.P., Podea D., Micluția I., Tiugan A., Păcală B., Grecu I.G. (2017). Raloxifene Plus Antipsychotics Versus Placebo Plus Antipsychotics in Severely Ill Decompensated Postmenopausal Women with Schizophrenia or Schizoaffective Disorder. J. Clin. Psychiatry.

[125] Marx C.E., Keefe R.S.E., Buchanan R.W., Hamer R.M., Kilts J.D., Bradford D.W., Strauss J.L., Naylor J.C., Payne V.M., Lieberman J.A. (2009). Proof-of-Concept Trial with the Neurosteroid Pregnenolone Targeting Cognitive and Negative Symptoms in Schizophrenia. Neuropsychopharmacology.

[126] Marx C.E., Lee J., Subramaniam M., Rapisarda A., Bautista D.C.T., Chan E., Kilts J.D., Buchanan R.W., Wai E.P., Verma S. (2014). Proof-of-concept randomized controlled trial of pregnenolone in schizophrenia. Psychopharmacology.

[127] Magalhães P.V.S., Dean O., Andreazza A.C., Berk M., Kapczinski F. (2016). Antioxidant treatments for schizophrenia. Cochrane Database Syst. Rev..

[128] Ni Y.-F., Zhang W., Bao X.-F., Wang W., Song L., Jiang B. (2016). GM1 ganglioside reverses the cognitive deficits induced by MK801 in mice. Behav. Pharmacol..

[129] Pitsikas N. (2015). The role of nitric oxide donors in schizophrenia: Basic studies and clinical applications. Eur. J. Pharmacol..

[130] Merritt K., Catalan A., Cowley S., Demjaha A., Taylor M., McGuire P., Cooper R., Morrison P. (2020). Glyceryl trinitrate in first-episode psychosis unmedicated with antipsychotics: A randomised controlled pilot study. J. Psychopharmacol..

[131] Yolland C.O.B., Phillipou A., Castle D.J., Neill E., Hughes M.E., Galletly C., Smith Z.M., Francis P.S., Dean O.M., Sarris J. (2020). Improvement of cognitive function in schizophrenia with N-acetylcysteine: A theoretical review. Nutr. Neurosci..

[132] Rapado-Castro M., Dodd S., Bush A.I., Malhi G.S., Skvarc D.R., On Z.X., Berk M., Dean O.M. (2017). Cognitive effects of adjunctive N -acetyl cysteine in psychosis. Psychol. Med..

[133] Ben-Azu B., Omogbiya I.A., Aderibigbe A.O., Umukoro S., Ajayi A.M., Iwalewa E.O. (2018). Doxycycline prevents and reverses schizophrenic-like behaviors induced by ketamine in mice via modulation of oxidative, nitrergic and cholinergic pathways. Brain Res. Bull..

[134] Kurita M., Holloway T., González-Maeso J. (2013). HDAC2 as a new target to improve schizophrenia treatment. Expert Rev. Neurother..

[135] Hai D., Shi S., Luo H. (2019). The therapeutic effect of quetiapine on cognitive impairment associated with 5-HT1A presynaptic receptor involved schizophrenia. J. Integr. Neurosci..

[136] Casey A.B., Canal C.E. (2017). Classics in Chemical Neuroscience: Aripiprazole. ACS Chem. Neurosci..

[137] Meller E., Goldstein M., Bohmaker K. (1990). Receptor reserve for 5-hydroxytryptamine1A-mediated inhibition of serotonin synthesis: Possible relationship to anxiolytic properties of 5-hydroxytryptamine1A agonists. Mol. Pharmacol..

[138] Sumiyoshi T., Matsui M., Nohara S., Yamashita I., Kurachi M., Sumiyoshi C., Jayathilake K., Meltzer H.Y. (2001). Enhancement of Cognitive Performance in Schizophrenia by Addition of Tandospirone to Neuroleptic Treatment. Am. J. Psychiatry.

[139] Rënyi L., Evenden J.L., Fowler C.J., Jerning E., Kelder D., Lake-Bakaar D., Larsson L.G., Mohell N., Sällemark M., Ross S.B. (2001). The pharmacological profile of (R)-3,4-dihydro-N-isopropyl-3-(N-isopropyl-N-propylamino)-2H-1-benzopyran-5-carboxamide, a selective 5-hydroxytryptamine(1A) receptor agonist. J. Pharmacol. Exp. Ther..

[140] Poddar I., Callahan P.M., Hernandez C.M., Yang X., Bartlett M.G., Terry A.V. (2018). Tropisetron enhances recognition memory in rats chronically treated with risperidone or quetiapine. Biochem. Pharmacol..

[141] Nikiforuk A. (2014). The procognitive effects of 5-HT6 receptor ligands in animal models of schizophrenia. Rev. Neurosci..

[142] Ivachtchenko A.V., Okun I., Aladinskiy V., Ivanenkov Y., Koryakova A., Karapetyan R., Mitkin O., Salimov R., Ivashchenko A., Bezprozvanny I. (2017). AVN-492, A Novel Highly Selective 5-HT6R Antagonist: Preclinical Evaluation. J. Alzheimer’s Dis..

[143] Zareifopoulos N., Papatheodoropoulos C. (2016). Effects of 5-HT-7 receptor ligands on memory and cognition. Neurobiol. Learn. Mem..

[144] Ohmura Y., Yoshida T., Konno K., Minami M., Watanabe M., Yoshioka M. (2015). Serotonin 5-HT 7 Receptor in the Ventral Hippocampus Modulates the Retrieval of Fear Memory and Stress-Induced Defecation. Int. J. Neuropsychopharmacol..

[145] Wang L., Zhang Y., Wang C., Zhang X., Wang Z., Liang X., Alachkar A., Civelli O. (2019). A Natural Product with High Affinity to Sigma and 5-HT7 Receptors as Novel Therapeutic Drug for Negative and Cognitive Symptoms of Schizophrenia. Neurochem. Res..

[146] Nikiforuk A., Kos T., Fijał K., Hołuj M., Rafa D., Popik P. (2013). Effects of the selective 5-HT7 receptor antagonist SB-269970 and amisulpride on ketamine-induced schizophrenia-like deficits in rats. PLoS ONE.

[147] Rajagopal L., Massey B.W., Michael E., Meltzer H.Y. (2016). Serotonin (5-HT)1A receptor agonism and 5-HT7 receptor antagonism ameliorate the subchronic phencyclidine-induced deficit in executive functioning in mice. Psychopharmacology.

[148] Nikiforuk A., Hołuj M., Kos T., Popik P. (2016). The effects of a 5-HT 5A receptor antagonist in a ketamine-based rat model of cognitive dysfunction and the negative symptoms of schizophrenia. Neuropharmacology.

[149] Yamazaki M., Harada K., Yamamoto N., Yarimizu J., Okabe M., Shimada T., Ni K., Matsuoka N. (2014). ASP5736, a novel 5-HT5A receptor antagonist, ameliorates positive symptoms and cognitive impairment in animal models of schizophrenia. Eur. Neuropsychopharmacol..

[150] Yamazaki M., Yamamoto N., Yarimizu J., Okabe M., Moriyama A., Furutani M., Marcus M.M., Svensson T.H., Harada K. (2018). Functional mechanism of ASP5736, a selective serotonin 5-HT5A receptor antagonist with potential utility for the treatment of cognitive dysfunction in schizophrenia. Eur. Neuropsychopharmacol..

[151] Casarotto P.C., Girych M., Fred S.M., Kovaleva V., Moliner R., Enkavi G., Biojone C., Cannarozzo C., Sahu M.P., Kaurinkoski K. (2021). Antidepressant drugs act by directly binding to TRKB neurotrophin receptors. Cell.

[152] Timić Stamenić T., Joksimović S., Biawat P., Stanković T., Marković B., Cook J.M., Savić M.M. (2015). Negative modulation of α 5 GABA A receptors in rats may partially prevent memory impairment induced by MK-801, but not amphetamine- or MK-801-elicited hyperlocomotion. J. Psychopharmacol..

[153] Arai S., Takuma K., Mizoguchi H., Ibi D., Nagai T., Kamei H., Kim H.-C., Yamada K. (2009). GABAB receptor agonist baclofen improves methamphetamine-induced cognitive deficit in mice. Eur. J. Pharmacol..

[154] Nudelman A., Gil-Ad I., Shpaisman N., Terasenko I., Ron H., Savitsky K., Geffen Y., Weizman A., Rephaeli A. (2008). A Mutual Prodrug Ester of GABA and Perphenazine Exhibits Antischizophrenic Efficacy with Diminished Extrapyramidal Effects. J. Med. Chem..

[155] Geffen Y., Nudelman A., Gil-Ad I., Rephaeli A., Huang M., Savitsky K., Klapper L., Winkler I., Meltzer H.Y., Weizman A. (2009). BL-1020: A novel antipsychotic drug with GABAergic activity and low catalepsy, is efficacious in a rat model of schizophrenia. Eur. Neuropsychopharmacol..

[156] Sadek B., Saad A., Sadeq A., Jalal F., Stark H. (2016). Histamine H3 receptor as a potential target for cognitive symptoms in neuropsychiatric diseases. Behav. Brain Res..

[157] Nirogi R., Grandhi V.R., Medapati R.B., Ganuga N., Benade V., Gandipudi S., Manoharan A., Abraham R., Jayarajan P., Bhyrapuneni G. (2021). Histamine 3 receptor inverse agonist Samelisant (SUVN-G3031): Pharmacological characterization of an investigational agent for the treatment of cognitive disorders. J. Psychopharmacol..

[158] Sagud M., Mihaljevic A., Pivac N. (2019). Smoking in schizophrenia: Recent findings about an old problem. Curr. Opin. Psychiatry.

[159] Boggs D.L., Carlson J., Cortes-Briones J., Krystal J.H., D’Souza D.C. (2014). Going up in smoke? A review of nAChRs-based treatment strategies for improving cognition in schizophrenia. Curr. Pharm. Des..

[160] AhnAllen C.G., Bidwell L.C., Tidey J.W. (2015). Cognitive effects of very low nicotine content cigarettes, with and without nicotine replacement, in smokers with schizophrenia and controls. Nicotine Tob. Res. Off. J. Soc. Res. Nicotine Tob..

[161] Verma M.K., Goel R.N., Bokare A.M., Dandekar M.P., Koul S., Desai S., Tota S., Singh N., Nigade P.B., Patil V.B. (2021). LL-00066471, a novel positive allosteric modulator of α7 nicotinic acetylcholine receptor ameliorates cognitive and sensorimotor gating deficits in animal models: Discovery and preclinical characterization. Eur. J. Pharmacol..

[162] Bristow L.J., Easton A.E., Li Y.-W., Sivarao D.V., Lidge R., Jones K.M., Post-Munson D., Daly C., Lodge N.J., Gallagher L. (2016). The Novel, Nicotinic Alpha7 Receptor Partial Agonist, BMS-933043, Improves Cognition and Sensory Processing in Preclinical Models of Schizophrenia. PLoS ONE.

[163] Beinat C., Banister S.D., Herrera M., Law V., Kassiou M. (2015). The Therapeutic Potential of α7 Nicotinic Acetylcholine Receptor (α7 nAChR) Agonists for the Treatment of the Cognitive Deficits Associated with Schizophrenia. CNS Drugs.

[164] Stoiljkovic M., Kelley C., Nagy D., Leventhal L., Hajós M. (2016). Selective activation of α7 nicotinic acetylcholine receptors augments hippocampal oscillations. Neuropharmacology.

[165] Huang M., Felix A.R., Flood D.G., Bhuvaneswaran C., Hilt D., Koenig G., Meltzer H.Y. (2014). The novel α7 nicotinic acetylcholine receptor agonist EVP-6124 enhances dopamine, acetylcholine, and glutamate efflux in rat cortex and nucleus accumbens. Psychopharmacology.

[166] Smith R.C., Amiaz R., Si T.-M., Maayan L., Jin H., Boules S., Sershen H., Li C., Ren J., Liu Y. (2016). Varenicline Effects on Smoking, Cognition, and Psychiatric Symptoms in Schizophrenia: A Double-Blind Randomized Trial. PLoS ONE.

[167] Potasiewicz A., Golebiowska J., Popik P., Nikiforuk A. (2018). Procognitive effects of varenicline in the animal model of schizophrenia depend on α4β2- and α 7-nicotinic acetylcholine receptors. J. Psychopharmacol..

[168] Terry A.V.J., Plagenhoef M., Callahan P.M. (2016). Effects of the nicotinic agonist varenicline on the performance of tasks of cognition in aged and middle-aged rhesus and pigtail monkeys. Psychopharmacology.

[169] Rook J.M., Bertron J.L., Cho H.P., Garcia-Barrantes P.M., Moran S.P., Maksymetz J.T., Nance K.D., Dickerson J.W., Remke D.H., Chang S. (2018). A Novel M(1) PAM VU0486846 Exerts Efficacy in Cognition Models without Displaying Agonist Activity or Cholinergic Toxicity. ACS Chem. Neurosci..

[170] Popiolek M., Mandelblat-Cerf Y., Young D., Garst-Orozco J., Lotarski S.M., Stark E., Kramer M., Butler C.R., Kozak R. (2019). In Vivo Modulation of Hippocampal Excitability by M4 Muscarinic Acetylcholine Receptor Activator: Implications for Treatment of Alzheimer’s Disease and Schizophrenic Patients. ACS Chem. Neurosci..

[171] Montani C., Canella C., Schwarz A.J., Li J., Gilmour G., Galbusera A., Wafford K., Gutierrez-Barragan D., McCarthy A., Shaw D. (2021). The M1/M4 preferring muscarinic agonist xanomeline modulates functional connectivity and NMDAR antagonist-induced changes in the mouse brain. Neuropsychopharmacology.

[172] Conley R.R., Boggs D.L., Kelly D.L., McMahon R.P., Dickinson D., Feldman S., Ball M.P., Buchanan R.W. (2009). The Effects of Galantamine on Psychopathology in Chronic Stable Schizophrenia. Clin. Neuropharmacol..

[173] Zhu W., Zhang Z., Qi J., Liu F., Chen J., Zhao J., Guo X. (2014). Adjunctive treatment for cognitive impairment in patients with chronic schizophrenia: A double-blind, placebo-controlled study. Neuropsychiatr. Dis. Treat..

[174] Koola M.M., Buchanan R.W., Pillai A., Aitchison K.J., Weinberger D.R., Aaronson S.T., Dickerson F.B. (2014). Potential role of the combination of galantamine and memantine to improve cognition in schizophrenia. Schizophr. Res..

[175] Koola M.M. (2018). Potential Role of Antipsychotic-Galantamine-Memantine Combination in the Treatment of Positive, Cognitive, and Negative Symptoms of Schizophrenia. Mol. Neuropsychiatry.

[176] Gawai P., Upadhyay R., Gakare S.G., Sarode L., Dravid S.M., Ugale R.R. (2020). Antipsychotic-like profile of CIQ isomers in animal models of schizophrenia. Behav. Pharmacol..

[177] Okada M., Fukuyama K., Kawano Y., Shiroyama T., Ueda Y. (2019). Memantine protects thalamocortical hyper-glutamatergic transmission induced by NMDA receptor antagonism via activation of system xc. Pharmacol. Res. Perspect..

[178] Javitt D.C. (2009). Glycine transport inhibitors for the treatment of schizophrenia: Symptom and disease modification. Curr. Opin. Drug Discov. Develop..

[179] Fone K.C.F., Watson D.J.G., Billiras R.I., Sicard D.I., Dekeyne A., Rivet J.-M., Gobert A., Millan M.J. (2020). Comparative Pro-cognitive and Neurochemical Profiles of Glycine Modulatory Site Agonists and Glycine Reuptake Inhibitors in the Rat: Potential Relevance to Cognitive Dysfunction and Its Management. Mol. Neurobiol..

[180] Goff D.C. (2017). D-cycloserine in Schizophrenia: New Strategies for Improving Clinical Outcomes by Enhancing Plasticity. Curr. Neuropharmacol..

[181] Mateo Z., Porter J.T. (2007). Group II metabotropic glutamate receptors inhibit glutamate release at thalamocortical synapses in the developing somatosensory cortex. Neuroscience.

[182] Lins B.R., Howland J.G. (2016). Effects of the metabotropic glutamate receptor 5 positive allosteric modulator CDPPB on rats tested with the paired associates learning task in touchscreen-equipped operant conditioning chambers. Behav. Brain Res..

[183] Sokolenko E., Hudson M.R., Nithianantharajah J., Jones N.C. (2019). The mGluR(2/3) agonist LY379268 reverses NMDA receptor antagonist effects on cortical gamma oscillations and phase coherence, but not working memory impairments, in mice. J. Psychopharmacol..

[184] Clifton N.E., Morisot N., Girardon S., Millan M.J., Loiseau F. (2013). Enhancement of social novelty discrimination by positive allosteric modulators at metabotropic glutamate 5 receptors: Adolescent administration prevents adult-onset deficits induced by neonatal treatment with phencyclidine. Psychopharmacology.

[185] Xing B., Han G., Wang M.-J., Snyder M.A., Gao W.-J. (2018). Juvenile treatment with mGluR2/3 agonist prevents schizophrenia-like phenotypes in adult by acting through GSK3β. Neuropharmacology.

[186] Cieślik P., Radulska A., Pelikant-Małecka I., Płoska A., Kalinowski L., Wierońska J.M. (2019). Reversal of MK-801-Induced Disruptions in Social Interactions and Working Memory with Simultaneous Administration of LY487379 and VU152100 in Mice. Int. J. Mol. Sci..

[187] Shen W., Plotkin J.L., Francardo V., Ko W.K.D., Xie Z., Li Q., Fieblinger T., Wess J., Neubig R.R., Lindsley C.W. (2015). M4 Muscarinic Receptor Signaling Ameliorates Striatal Plasticity Deficits in Models of L-DOPA-Induced Dyskinesia. Neuron.

[188] Chater T.E., Goda Y. (2014). The role of AMPA receptors in postsynaptic mechanisms of synaptic plasticity. Front. Cell. Neurosci..

[189] Chen S., Zhao Y., Wang Y., Shekhar M., Tajkhorshid E., Gouaux E. (2017). Activation and Desensitization Mechanism of AMPA Receptor-TARP Complex by Cryo-EM. Cell.

[190] Zheng Y., Balabhadrapatruni S., Masumura C., Darlington L.C., Smith P.F. (2011). Effects of the Putative Cognitive-Enhancing Ampakine, CX717, on Attention and Object Recognition Memory. Curr. Alzheimer Res..

[191] Bruce H.A., Kochunov P., Paciga S.A., Hyde C.L., Chen X., Xie Z., Zhang B., Xi H.S., O’Donnell P., Whelan C. (2017). Potassium channel gene associations with joint processing speed and white matter impairments in schizophrenia. Genes Brain Behav..

[192] Kozak R., Kiss T., Dlugolenski K., Johnson D.E., Gorczyca R.R., Kuszpit K., Harvey B.D., Stolyar P., Sukoff Rizzo S.J., Hoffmann W.E. (2020). Characterization of PF-6142, a Novel, Non-Catecholamine Dopamine Receptor D1 Agonist, in Murine and Nonhuman Primate Models of Dopaminergic Activation. Front. Pharmacol..

[193] Tanyeri P., Buyukokuroglu M.E., Mutlu O., Ulak G., Akar F.Y., Celikyurt I.K., Erden B.F. (2015). Effects of ziprasidone, SCH23390 and SB277011 on spatial memory in the Morris water maze test in naive and MK-801 treated mice. Pharmacol. Biochem. Behav..

[194] Zahrt J., Taylor J.R., Mathew R.G., Arnsten A.F.T. (1997). Supranormal stimulation of D1 dopamine receptors in the rodent prefrontal cortex impairs spatial working memory performance. J. Neurosci..

[195] Svensson K.A., Hao J., Bruns R.F., Witkin J.M. (2019). Chapter Nine—Positive allosteric modulators of the dopamine D1 receptor: A new mechanism for the treatment of neuropsychiatric disorders. Neuropsychotherapeutics.

[196] Wilbraham D., Biglan K.M., Svensson K.A., Tsai M., Kielbasa W. (2021). Safety, Tolerability, and Pharmacokinetics of Mevidalen (LY3154207), a Centrally Acting Dopamine D1 Receptor-Positive Allosteric Modulator (D1PAM), in Healthy Subjects. Clin. Pharmacol. Drug Dev..

[197] Hatzipantelis C.J., Langiu M., Vandekolk T.H., Pierce T.L., Nithianantharajah J., Stewart G.D., Langmead C.J. (2020). Translation-Focused Approaches to GPCR Drug Discovery for Cognitive Impairments Associated with Schizophrenia. ACS Pharmacol. Transl. Sci..

[198] Huang M., Kwon S., Oyamada Y., Rajagopal L., Miyauchi M., Meltzer H.Y. (2015). Dopamine D3 receptor antagonism contributes to blonanserin-induced cortical dopamine and acetylcholine efflux and cognitive improvement. Pharmacol. Biochem. Behav..

[199] Mutti V., Fiorentini C., Missale C., Bono F. (2020). Dopamine D3 receptor heteromerization: Implications for neuroplasticity and neuroprotection. Biomolecules.

[200] Manvich D.F., Petko A.K., Branco R.C., Foster S.L., Porter-Stransky K.A., Stout K.A., Newman A.H., Miller G.W., Paladini C.A., Weinshenker D. (2019). Selective D2 and D3 receptor antagonists oppositely modulate cocaine responses in mice via distinct postsynaptic mechanisms in nucleus accumbens. Neuropsychopharmacology.

[201] Torrisi S.A., Laudani S., Contarini G., De Luca A., Geraci F., Managò F., Papaleo F., Salomone S., Drago F., Leggio G.M. (2020). Dopamine, Cognitive Impairments and Second-Generation Antipsychotics: From Mechanistic Advances to More Personalized Treatments. Pharmaceuticals.

[202] Minzenberg M.J., Carter C.S. (2008). Modafinil: A Review of Neurochemical Actions and Effects on Cognition. Neuropsychopharmacology.

[203] Murillo-Rodríguez E., Barciela Veras A., Barbosa Rocha N., Budde H., Machado S. (2018). An Overview of the Clinical Uses, Pharmacology, and Safety of Modafinil. ACS Chem. Neurosci..

[204] Dawson N., Thompson R.J., McVie A., Thomson D.M., Morris B.J., Pratt J.A. (2012). Modafinil reverses phencyclidine-induced deficits in cognitive flexibility, cerebral metabolism, and functional brain connectivity. Schizophr. Bull..

[205] Rogóż Z., Kamińska K. (2016). The effect of combined treatment with escitalopram and risperidone on the MK-801-induced changes in the object recognition test in mice. Pharmacol. Rep..

[206] Bruno A., Zoccali R., Bellinghieri P.M., Pandolfo G., De Fazio P., Spina E., Muscatello M.R.A. (2014). Reboxetine adjuvant therapy in patients with schizophrenia showing a suboptimal response to clozapine: A 12-week, open-label, pilot study. J. Clin. Psychopharmacol..

[207] Bymaster F.P., Perry K.W., Tollefson G.D. (1998). Combination Therapy for Treatment of Psychoses.

[208] Al-Nema M.Y., Gaurav A. (2020). Phosphodiesterase as a Target for Cognition Enhancement in Schizophrenia. Curr. Top. Med. Chem..

[209] Duinen M., Reneerkens O., Lambrecht L., Sambeth A., Rutten B., Os J., Blokland A., Prickaerts J. (2015). Treatment of Cognitive Impairment in Schizophrenia: Potential Value of Phosphodiesterase Inhibitors in Prefrontal Dysfunction. Curr. Pharm. Des..

[210] Snyder G.L., Vanover K.E. (2017). PDE Inhibitors for the Treatment of Schizophrenia. Adv. Neurobiol..

[211] Enomoto T., Tatara A., Goda M., Nishizato Y., Nishigori K., Kitamura A., Kamada M., Taga S., Hashimoto T., Ikeda K. (2019). A novel phosphodiesterase 1 inhibitor DSR-141562 exhibits efficacies in animal models for positive, negative, and cognitive symptoms associated with schizophrenia. J. Pharmacol. Exp. Ther..

[212] Ahmed H.I., Abdel-Sattar S.A., Zaky H.S. (2018). Vinpocetine halts ketamine-induced schizophrenia-like deficits in rats: Impact on BDNF and GSK-3β/β-catenin pathway. Naunyn. Schmiedebergs. Arch. Pharmacol..

[213] O’Brien J.J., O’Callaghan J.P., Miller D.B., Chalgeri S., Wennogle L.P., Davis R.E., Snyder G.L., Hendrick J.P. (2020). Inhibition of calcium-calmodulin-dependent phosphodiesterase (PDE1) suppresses inflammatory responses. Mol. Cell. Neurosci..

[214] Millar K.J., Mackie S., Clapcote S.J., Murdoch H., Pickard B.S., Christie S., Muir W.J., Blackwood D.H., Roder J.C., Houslay M.D. (2007). Disrupted in schizophrenia 1 and phosphodiesterase 4B: Towards an understanding of psychiatric illness. J. Physiol..

[215] Gilleen J., Nottage J., Yakub F., Kerins S., Valdearenas L., Uz T., Lahu G., Tsai M., Ogrinc F., Williams S.C. (2021). The effects of roflumilast, a phosphodiesterase type-4 inhibitor, on EEG biomarkers in schizophrenia: A randomised controlled trial. J. Psychopharmacol..

[216] Zagorska A., Partyka A., Bucki A., Gawalskax A., Czopek A., Pawlowski M. (2018). Phosphodiesterase 10 Inhibitors—Novel Perspectives for Psychiatric and Neurodegenerative Drug Discovery. Curr. Med. Chem..

[217] Smith S.M., Uslaner J.M., Cox C.D., Huszar S.L., Cannon C.E., Vardigan J.D., Eddins D., Toolan D.M., Kandebo M., Yao L. (2013). The novel phosphodiesterase 10A inhibitor THPP-1 has antipsychotic-like effects in rat and improves cognition in rat and rhesus monkey. Neuropharmacology.

[218] Takakuwa M., Watanabe Y., Saijo T., Murata M., Anabuki J., Tezuka T., Sato S., Kojima K., Hashimoto K. (2020). Antipsychotic-like effects of a novel phosphodiesterase 10A inhibitor MT-3014 in rats. Pharmacol. Biochem. Behav..

[219] Yurgelun-Todd D.A., Renshaw P.F., Goldsmith P., Uz T., Macek T.A. (2020). A randomized, placebo-controlled, phase 1 study to evaluate the effects of TAK-063 on ketamine-induced changes in fMRI BOLD signal in healthy subjects. Psychopharmacology.

[220] Bradley A.J., Dinan T.G. (2010). A systematic review of hypothalamic-pituitary-adrenal axis function in schizophrenia: Implications for mortality. J. Psychopharmacol..

[221] Pitsikas N., Zoupa E., Gravanis A. (2021). The novel dehydroepiandrosterone (DHEA) derivative BNN27 counteracts cognitive deficits induced by the D1/D2 dopaminergic receptor agonist apomorphine in rats. Psychopharmacology.

[222] Soria V., González-Rodríguez A., Huerta-Ramos E., Usall J., Cobo J., Bioque M., Barbero J.D., García-Rizo C., Tost M., Monreal J.A. (2018). Targeting hypothalamic-pituitary-adrenal axis hormones and sex steroids for improving cognition in major mood disorders and schizophrenia: A systematic review and narrative synthesis. Psychoneuroendocrinology.

[223] Chakrabarti M., Haque A., Banik N.L., Nagarkatti P., Nagarkatti M., Ray S.K. (2014). Estrogen receptor agonists for attenuation of neuroinflammation and neurodegeneration. Brain Res. Bull..

[224] Bergemann N., Parzer P., Jaggy S., Auler B., Mundt C., Maier-Braunleder S. (2008). Estrogen and Comprehension of Metaphoric Speech in Women Suffering from Schizophrenia: Results of a Double-Blind, Placebo-Controlled Trial. Schizophr. Bull..

[225] Lobo R.A. (2017). Hormone-replacement therapy: Current thinking. Nat. Rev. Endocrinol..

[226] Marx C.E., Bradford D.W., Hamer R.M., Naylor J.C., Allen T.B., Lieberman J.A., Strauss J.L., Kilts J.D. (2011). Pregnenolone as a novel therapeutic candidate in schizophrenia: Emerging preclinical and clinical evidence. Neuroscience.

[227] Winship I.R., Dursun S.M., Baker G.B., Balista P.A., Kandratavicius L., Maia-de-Oliveira J.P., Hallak J., Howland J.G. (2019). An Overview of Animal Models Related to Schizophrenia. Can. J. Psychiatry.

[228] Bray N.J., Kapur S., Price J. (2012). Investigating schizophrenia in a “dish”: Possibilities, potential and limitations. World Psychiatry.

[229] Perkovic M., Erjavec G., Strac D., Uzun S., Kozumplik O., Pivac N. (2017). Theranostic Biomarkers for Schizophrenia. Int. J. Mol. Sci..

[230] Dobber J., Latour C., de Haan L., Scholte op Reimer W., Peters R., Barkhof E., van Meijel B. (2018). Medication adherence in patients with schizophrenia: A qualitative study of the patient process in motivational interviewing. BMC Psychiatry.

